# TRAF6 Phosphorylation Prevents Its Autophagic Degradation and Re-Shapes LPS-Triggered Signaling Networks

**DOI:** 10.3390/cancers13143618

**Published:** 2021-07-19

**Authors:** Julia Busch, Rita Moreno, Laureano de la Vega, Vera Vivian Saul, Susanne Bacher, Felix von Zweydorf, Marius Ueffing, Axel Weber, Christian Johannes Gloeckner, Uwe Linne, Michael Kracht, Michael Lienhard Schmitz

**Affiliations:** 1 Institute of Biochemistry, Member of the German Center for Lung Research, Justus Liebig University, 35392 Giessen, Germany; julia.busch@ddz.de (J.B.); Vera.Saul@biochemie.med.uni-giessen.de (V.V.S.); Susanne.Bacher@biochemie.med.uni-giessen.de (S.B.); 2 Division of Cellular Medicine, Ninewells Hospital and Medical School, University of Dundee, James Arrott Drive, Dundee DD1 9SY, UK; r.morenodorta@dundee.ac.uk (R.M.); l.delavega@dundee.ac.uk (L.d.l.V.); 3 German Center for Neurodegenerative Diseases (DZNE), 72076 Tübingen, Germany; felix.von-zweydorf@dzne.de (F.v.Z.); johannes.gloeckner@dzne.de (C.J.G.); 4 Centre for Ophthalmology, Institute for Ophthalmic Research, University of Tübingen, 72076 Tübingen, Germany; marius.ueffing@uni-tuebingen.de; 5 Rudolf Buchheim Institute of Pharmacology, Member of the German Center for Lung Research, Justus Liebig University, 35392 Giessen, Germany; axel.weber@pharma.med.uni-giessen.de (A.W.); Michael.Kracht@pharma.med.uni-giessen.de (M.K.); 6 Core Facility for Medical Bioanalytics, Center for Ophthalmology, Institute for Ophthalmic Research, University of Tübingen, 72076 Tübingen, Germany; 7 Mass Spectrometry Facility of the Department of Chemistry, Philipps University, 35043 Marburg, Germany; linneu@staff.uni-marburg.de

**Keywords:** NF-κB, TRAF6, IKKε, phosphorylation, kinase activation, signaling, autophagy, LPS

## Abstract

**Simple Summary:**

Here, we reveal that basal turnover and autophagy-induced decay of the ubiquitin E3 ligase TRAF6 is antagonized by IKKε-mediated phosphorylation at five serines. Phosphoproteomic experiments show that TRAF6 and its phosphorylation contribute to the remodeling of LPS- and autophagyinduced signaling networks, revealing an intricate link between inflammatory and metabolic processes that are frequently dysregulated in cancer.

**Abstract:**

The ubiquitin E3 ligase TNF Receptor Associated Factor 6 (TRAF6) participates in a large number of different biological processes including innate immunity, differentiation and cell survival, raising the need to specify and shape the signaling output. Here, we identify a lipopolysaccharide (LPS)-dependent increase in TRAF6 association with the kinase IKKε (inhibitor of NF-κB kinase subunit ε) and IKKε-mediated TRAF6 phosphorylation at five residues. The reconstitution of TRAF6-deficient cells, with TRAF6 mutants representing phosphorylation-defective or phospho-mimetic TRAF6 variants, showed that the phospho-mimetic TRAF6 variant was largely protected from basal ubiquitin/proteasome-mediated degradation, and also from autophagy-mediated decay in autolysosomes induced by metabolic perturbation. In addition, phosphorylation of TRAF6 and its E3 ligase function differentially shape basal and LPS-triggered signaling networks, as revealed by phosphoproteome analysis. Changes in LPS-triggered phosphorylation networks of cells that had experienced autophagy are partially dependent on TRAF6 and its phosphorylation status, suggesting an involvement of this E3 ligase in the interplay between metabolic and inflammatory circuits.

## 1. Introduction

Transmission and amplification of immune signaling employs a cascade of enzymatic reactions that lead to the regulated attachment of posttranslational modifications including ubiquitination and phosphorylation [1,2]. Ubiquitin signaling employs differentially composed ubiquitin chains including K48-branched ubiquitin chains, which mediate the ubiquitin/proteasome-dependent degradation of target proteins, and K63-branched polyubiquitin chains, which are recognized and bound by chain readers such as TAB2/TAB3 (TGF-ß activated kinase 1/MAP3K7 binding protein 2/3) [3]. Ubiquitin is attached to substrate proteins by consecutive reactions employing E1 and E2 enzymes, with the last step executed by an ubiquitin E3 ligase [4]. A well-studied E3 ligase with relevance to immune cell signaling is TRAF6, which typically promotes the formation of K63-linked polyubiquitin chains. TRAF6 contains a so-called RING (really interesting new gene) domain, is ubiquitously expressed and transduces signals from various immune receptors including those of the tumor necrosis factor receptor (TNF-R) superfamily, interleukin 1 (IL-1) receptors, T-cell receptors and many toll-like receptors (TLRs) including the LPS-responsive TLR4 [5]. Extracellular LPS leads to TLR4-dependent signaling and inducible receptor association of TRAF6, which, in turn, allows the interaction of TRAF6 with the E2 ubiquitin-conjugating enzyme complex (Ubc13-Uev1A) to catalyze the formation of K63-linked polyubiquitination on itself or its substrate proteins [6]. TRAF6-mediated K63 ubiquitination of its client proteins enables interaction with the ubiquitin-binding domains of adapter proteins such as TAB2/TAB3, which then leads to activation of downstream kinases including TGFβ-activated kinase 1 (TAK1) [7,8]. Increased TAK1 activity triggers the activation of proinflammatory mitogen-activated protein kinases (MAPKs) [9], thus coupling ubiquitination to multiple downstream phosphorylation networks. TRAF6 is controlled by several mechanisms that include inducible protein/protein interactions, allowing its docking to surface receptors or downstream effectors. In addition, the expression levels of TRAF6 are supervised by microRNAs such as miR-146a and long noncoding RNAs [10,11]. Furthermore, TRAF6 levels are restricted by ubiquitin/proteasome-mediated decay or elimination in the autophagosome [12,13].

TRAF6 regulates not only immune signaling, but also further processes including tumor cell growth [14], genome integrity [15], gene-specific mRNA decay [16] as well as osteoclast differentiation [17]. TRAF6 also contributes to early steps in the process of autophagy, which is induced in response to stress stimuli including growth factor depletion, nutrient deprivation and infection. Autophagy serves to eliminate unwanted and potentially harmful cytosolic material, such as protein aggregates or damaged organelles, to provide nutrients for vital cellular functions during fasting and other forms of stress, including infection [18,19]. TRAF6 contributes to the early steps of autophagy and promotes the formation of phagophores that engulf cytosolic materials for degradation and recycling. In this process, TRAF6 promotes phagophore formation through the K63-linked polyubiquitination of ULK1 (Unc-51 Like Autophagy Activating Kinase 1) and BECLIN-1 (Coiled-Coil Myosin-Like BCL2-Interacting Protein) [20,21]. Phagophores further mature to a double-membraned vesicle termed autophagosome, which then fuses with the lysosomal membrane to form autolysosomes, the sites where acidic hydrolases degrade the autophagic cargo, including the TRAF6 interactor and autophagy receptor p62, also known as Sequestosome-1 (SQSTM1) [22,23].

The process of autophagy shows many levels of cross-regulation with innate and adaptive immunity [24]. Infection with viruses or bacteria frequently triggers autophagy, where the invaders, such as Salmonella, are trapped within an autophagosome and targeted to the lysosome for destruction [25]. These principal defence mechanisms may also explain why autophagy is also induced upon stimulation of various immune receptors including TLR4 [26,27]. *Vice versa*, autophagy facilitates the TLR4-induced ubiquitination of TRAF6 and activation of NF-κB, leading to increased production of cytokines [28]. The intimate relation between autophagy and inflammation is also witnessed by the dual function of proteins with a role in inflammatory processes and also autophagy, as exemplified by TRAF6 and the kinases IKKε and ULK1/2 [29,30,31].

IKKε contributes to the type I interferon expression in response to viral infections, as it phosphorylates and, thus, activates IRF3/7 (interferon regulatory factor 3/7) transcription factors [32]. Furthermore, IKKε overexpression contributes to the development of breast cancer and KRAS-induced pancreas tumors [33,34]. IKKε activity is regulated by autocatalytic phosphorylation at the activation loop, while its intracellular localization is controlled by attachment of the small ubiquitin-like modifier (SUMO) peptide to Lys 231 in response to DNA damage [35].

Here, we reveal an increased association between IKKε and TRAF6 in LPS-stimulated cells and the IKKε-mediated phosphorylation of TRAF6 at five serine residues. These phosphorylation events serve at least two functions, as they lead to the stabilization of TRAF6 under basal conditions and in response to inducers of autophagy. In addition, basal and stimulus-induced TRAF6 phosphorylations specify the downstream signaling networks, as determined by phosphoproteomic analysis.

## 2. Materials and Methods

### 2.1. Cell Culture and CRISPR-Cas9-Mediated Generation of TRAF6 Knockout Cells

HeLa, HEK293 and 293 TLR4 cells were cultured in DMEM (Life Technologies, Carlsbad, CA, USA) supplemented with 10% FCS, 100 U/mL penicillin and 100 µg/mL streptomycin. A total of 293 TLR4 cells were cultured in the presence of 1 mg/mL G418. For CRISPR-Cas9 mediated knockout of TRAF6, 1000 293 TLR4 cells per 10-cm dish were transfected with 4 µg of the px459 vector containing a sgRNA targeting the first exon of TRAF6. One day later, the non-transfected cells were eliminated by the addition of puromycin (2 µg/mL) for 48 h. Single cell-derived clones were picked after approximately one week and further analyzed for the expression of TRAF6 and Cas9.

### 2.2. Characterization of CRISPR-Cas9-Mediated Genomic Indel Mutations

Genomic DNA of cells deficient in TRAF6 protein expression was isolated using the NucleoSpin Tissue Kit (Macherey-Nagel, Düren, Germany). A region containing the TRAF6 locus with the expected Indel mutation was PCR amplified using the Phusion High Fidelity DNA polymerase (ThermoFisher Scientific, Waltham, MA, USA), 50 ng of genomic DNA and TRAF6 intron-exon-spanning oligonucleotides. A PCR was performed in a thermocycler with 30 amplification cycles and the purified PCR product was sequenced using one of the PCR primers as a sequencing primer.

### 2.3. Induction of Autophagy

Cells that reached approximately 60% confluence were washed twice with PBS and incubated in EBSS medium (140 mM NaCl; 1 mM CaCl_2_; 1 mM MgCl_2_; 5 mM glucose; 20 mM HEPES (pH 7.4); 1% BSA—sterile filtrated using a 0.2 µm filter) to induce autophagy, as described previously [35]. Cells were further cultivated at 37 °C and 5% (*v*/*v*) CO_2_ for various periods and then harvested for further analysis.

### 2.4. Cell Transfection and Generation of Stable Cell Lines

Cells were seeded in 10-cm dishes 24 h before transfection. A desired amount of plasmid DNA was mixed with 200 µL of serum-free DMEM without antibiotics. Furthermore, 2 µL of linear Polyethyleneimine (PEI, 1 mg/mL H_2_O) per µg plasmid DNA was mixed with serum-free DMEM without antibiotics and added to the premixed DNA to reach a total volume of 400 µL. After an incubation of 20 min at room temperature, the solution was transferred to the cells. The medium of those cells was exchanged with 5 mL antibiotic-free medium with normal FCS concentration. Cells were then incubated for 4 h and the medium was replaced with complete medium. In order to generate 293 TLR4 cells that stably express the various HA-TRAF6 variants, TRAF6 knockout cells were transfected with vectors encoding the WT and mutated TRAF6 proteins, followed by selection with 2 µg/mL puromycin for one week. Individual cell colonies were picked and analyzed by Western blotting for TRAF6 expression levels. Only cell clones with comparable TRAF6 levels were used for the analysis; all key experiments were conducted with several independent clones or cell pools in order to ensure that the effects are not due to clonal variations.

### 2.5. Lambda Protein Phosphatase Assays

To test whether the slower migration of proteins in gel electrophoresis is caused by phosphorylation, lysates were treated with the Mn^2+^-dependent λ-phosphatase with activity towards all phosphorylated amino acids. For this purpose, cells were lysed under native conditions in IGEPAL buffer lacking phosphatase inhibitors and cleared supernatants were mixed with 400 units of λ-phosphatase, supplemented with 2 mM MnCl_2_ and incubated at 30 °C for 20 min. Samples were then mixed with 5 × SDS sample buffer and heated for 4 min at 95 °C for further Western blot analysis.

### 2.6. Denaturing Lysis and Enrichment of His-Tagged Proteins on Ni-NTA Columns

His6-Ubiquitin-modified proteins expressed in eukaryotic cells were enriched after lysis under denaturing conditions using Ni-NTA agarose beads (Qiagen, Hilden, Germany). The cells were collected by centrifugation and 1/10 of the cells were used as input control and lysed using 1 × SDS sample buffer. The remaining cells were lysed in 650 µL Gu-HCl lysis buffer (6 M Guanidine-HCl, 0.1 M Na_2_HPO_4_/NaH_2_PO_4_, 10 mM Tris-Base, 10 mM Imidazole, 1 mM ß-Mercaptoethanol pH = 8.0). The DNA was sheared by sonification and lysates were cleared by centrifugation (10 min, 16,000× *g*). The supernatant was mixed with 60 µL of equilibrated Ni-NTA agarose, followed by incubation for at least 4 h at room temperature under agitation. Subsequently, the beads were serially washed in different Urea wash buffers, as described previously [36], and the bound proteins were eluted by boiling the beads for 5 min in 50 µL 2.5 × SDS sample buffer containing 200 mM imidazole. The purified His6-Ubiquitin-modified proteins were further analyzed by immunoblotting.

### 2.7. Native Cell Lysis and Co-Immunoprecipitation

Cells were washed first with cold 1 × PBS, harvested by scraping in 1 mL of PBS and then transferred to reaction tubes. After centrifugation (300× *g*, 5 min), the cell pellet was resuspended in IGEPAL lysis buffer (20 mM Tris/HCl, pH 7.5, 150 mM NaCl, 1% (*v*/*v*) IGEPAL CA-630, freshly added: 20 mM NaF, 1 mM Na_3_VO_4_, 1 mM PMSF, 4 µg/mL aprotinin, 4 µg/mL leupeptin, 0.5 mM PMSF) and incubated for 20 min on ice. Cell debris was cleared by centrifugation (16,000× *g*, 10 min); the supernatants were transferred to a new tube and either used for co-immunoprecipitation experiments or mixed with 5 × SDS sample buffer, boiled at 95 °C for 5 min and analyzed by Western blotting. Co-immunoprecipitation was performed by pre-clearing of the lysates by the addition of 15 µL of A/G-agarose bead slurry and incubation for 1 h at 4 °C under agitation. After centrifugation (5 min, 2000× *g*), the cleared lysate was transferred to a fresh tube. In total, 10% of lysate was used for the input control and the rest was adjusted to a volume of 500 µL with IGEPAL lysis buffer. Quantities of 1 µg of the immunoprecipitating antibody or control IgG antibody and 25 µL of protein A/G agarose were added. The samples were incubated for 4 h at 4 °C on a rotating spinning wheel. The beads were washed five times with 1 mL of cold IGEPAL buffer by inverting the tube, followed by centrifugation (1 min, 2000× *g*). Proteins were eluted from the beads with 1.5 × SDS sample buffer and analyzed by Western blotting as described previously [31].

### 2.8. Mass Spectrometry for Detection of TRAF6 Phosphorylation Sites

293T cells were transfected to express Flag-tagged IKKε and Flag-tagged TRAF6. The next day, cells were lysed in IGEPAL lysis buffer containing phosphatase inhibitors, followed by immunoprecipitation of TRAF6 and IKKε using anti-FLAG antibodies. The proteins were eluted by incubation with SDS sample buffer and subjected to SDS-PAGE. Gels were stained by Coomassie Brilliant-Blue G-250 and bands corresponding to TRAF6 were excised and subjected to proteomic analysis.

### 2.9. Phospho-Proteomic Analysis of TRAF6-Mediated Signaling Pathways

HEK293 TLR4 cells stably expressing TRAF6 WT and its mutants were grown to 70% confluence, followed by treatment and LPS stimulation as specified in the figure legends. After treatment, cells were washed with cold PBS and lysed in Urea lysis buffer (20 mM HEPES (pH 8.0), 9.0 M urea, 1 mM activated Na_3_VO_4_, 2.5 mM Na_4_P_2_O_7_, 1 mM ß-glycerol-phosphate). Samples were sonicated and centrifuged for 20 min at 4 °C at 16,100× *g*. The supernatants were collected, and the protein concentration was determined using a Bradford assay. An aliquot was used for control Western blots, ensuring the successful cell treatment, while the remaining material was used for mass spectrometry. Samples containing 2 mg total protein each dissolved in 8 M urea were reduced with 5 mM TCEP for 1 h at 37 °C/1000 rpm, and carbamidomethylated by incubating the samples with 10 mM iodoacetamide at 500 rpm for 30 min at room temperature in the dark. Subsequently, N-Acetyl-cystein was added to a final concentration of 12.5 mM. Samples were diluted to 6 M urea through the addition of 0.1 M NH_4_HCO_3_. LysC was added at a concentration of 1:200 in relation to the total protein content (*w*/*w*) and samples were incubated for 3–4 h at 37 °C. Prior to the addition of sequencing grade trypsin (1:50 (*w*/*w*)), samples were further diluted to a urea concentration of 1.6 M using 0.1 M NH_4_HCO_3_. After incubating samples overnight at 37 °C, the samples were acidified using TFA and peptides were desalted and concentrated using Chromabond C18WP spin columns (Macherey-Nagel, Part No. 730522). Finally, peptides were dissolved in 25 µL of water with 5% acetonitrile and 0.1% formic acid. Phosphopeptide enrichment was performed with MagReSyn Ti-IMAC magnetic beads (ReSyn Biosciences, Edenvale, South Africa) according to the manufacturer’s protocols. Finally, samples were dissolved 25 µL 5% MeCN/0.1% TFA. The mass spectrometric analysis of the samples was performed using an Orbitrap Velos Pro mass spectrometer (ThermoFisher Scientific, Waltham, MA, USA). An Ultimate nanoRSLC-HPLC system (Dionex, Sunnyvale, CA, USA), equipped with a custom end-fritted 50 cm × 75 µm C18 RP column filled with 2.4 µm beads, was connected online to the mass spectrometer through a Proxeon nanospray source. Then, 6 µL of the tryptic digest were injected onto a 300 µm ID × 1 cm C18 PepMap pre-concentration column (Thermo Scientific). Automated trapping and desalting of the sample were performed at a flowrate of 6 µL/min using water/0.05% formic acid as solvent. Separation of the tryptic peptides was achieved with the following gradient of water/0.05% formic acid (solvent A) and 80% acetonitrile/0.045% formic acid (solvent B) at a flow rate of 300 nL/min: holding 4% B for five minutes, followed by a linear gradient to 45% B within 30 min and linear increase to 95% solvent B for an additional 5 min. The column was connected to a stainless steel nanoemitter (Proxeon, Odense, Denmark) and the eluent was sprayed directly towards the heated capillary of the mass spectrometer using a potential of 2300 V. A survey scan with a resolution of 60,000 within the Orbitrap mass analyzer was combined with twenty data-dependent MS/MS scans with dynamic exclusion for 30 s using CID with the linear ion-trap and multistage activation enabled. For better data reliability, a technical replicate was measured for all samples. Data analysis was performed using Proteome Discoverer 2.2 (Thermo Scientific) with the SEQUEST search engine (Accessed on 18 August 2020) or MaxQuant (Version 1.5.7.4) with the Andromeda (Accessed on 18 August 2020) search engine.

### 2.10. LC-MS/MS Analysis

For the identification of TRAF6 phosphorylation sites, SDS-PAGE gel bands corresponding to TRAF6 in the absence or presence of IKKε have been excised and processed by tryptic in-gel proteolysis following standard protocols [37]. Extracted and dried peptides were re-dissolved in 0.5% TFA and subjected to LC-MSMS analysis on a nanoflow HPLC system (Ultimate 3000 RSLC; Thermo Fisher) coupled to an Orbitrap Velos (Thermo Fisher) tandem mass spectrometer using 120 min gradients for the C-18 reverse chromatography and a TOP10 method combined with CID fractionation for MSMS. In addition, for evidence of the exact phospho-site, multi-stage activation has been used. For database search, tandem mass spectra were extracted by MSConvert (version year 2010). Charge state deconvolution and deisotoping were not performed. All MS/MS samples were analyzed using Mascot (Matrix Science, version 2.3). Mascot was set up to search the SwissProt database (selected for Homo sapiens, 2010_12, 20,259 entries, accessed on 30 October 2010) assuming the digestion enzyme trypsin. Mascot was searched with a fragment ion mass tolerance of 0.60 Da and a parent ion tolerance of 10.0 PPM. Carbamidomethyl of cysteine was specified in Mascot as a fixed modification. Deamidation of asparagine and glutamine, and oxidation of methionine and phospho-serine/threonine/tyrosine were specified in Mascot as variable modifications. The search engine results/spectra were analyzed and visualized by Scaffold (Proteome Software, version 3_00_8).

### 2.11. Statistical Rationale and Bioinformatic Analysis

The protein signals from Western blots were detected using the ChemiDocTM XRS+ System (Bio-Rad Laboratories, Hercules, CA, USA) and further processed using Image Lab Software. For the data shown in Figures 6–8 and Figure S6, raw data from 82 LC-MS/MS runs (representing three independent experiments and two technical replicates per sample, except for samples 322 (P-def + EBSS + LPS) and 332 (P-mim. untreated), which had only one sample measured (with no technical replicate)) were mapped to the Homo sapiens proteome (uniprot ID UP000005640). All data sets were processed by MaxQuant (version 1.6.14.0 [38], with the match between runs option enabled), resulting in the identification of 17,220 human protein IDs that corresponded to phosphorylated peptides. The log_2_-transformed phosphopeptide intensities were width normalized and log_2_ transformed with Perseus 1.6.14.0 [39]. IDs assigned to contaminants and reverse sequences were omitted. The 3 × 2 replicates from each condition were assigned to one analysis group and, for further calculations and for comparisons between treatment groups by two sample Student’s T-tests, 2835 phosphopeptides with at least 5 intensity values per peptide in at least one group were selected. From this group, differentially expressed phosphopeptides were identified from log_2_ transformed normalized protein intensity values by T-test analysis based on a *p*-value ≤ −log_10_ of 1.3. In addition, phosphopeptides only found in one out of two pairwise comparisons were considered as regulated and added to the total number of differentially regulated phosphopeptides per condition. Subsequent filtering steps were performed in Excel 2016 according to criteria described in the figure legends. Graphical representations were generated with GrapPad Prism 8.4.3. Venn diagrams were created with tools provided at http://bioinformatics.psb.ugent.be/webtools/Venn/, accessed on 18 May 2021. Overrepresentation analyses of gene sets were conducted using majority protein IDs or gene IDs that corresponded to differentially enriched phosphopeptides using Metascape software (https://metascape.org/, accessed on 18 May 2021) with the express settings [40]. Clustered enrichment maps show the top 20 pathways and the enrichment *p*-values.

### 2.12. Antibodies, Plasmids and Reagents

This information is given in Table S1.

## 3. Results

### 3.1. IKKε Shows LPS-Triggered Interaction with TRAF6 and Phosphorylates TRAF6 at Five Different Serines

**Figure 1:**
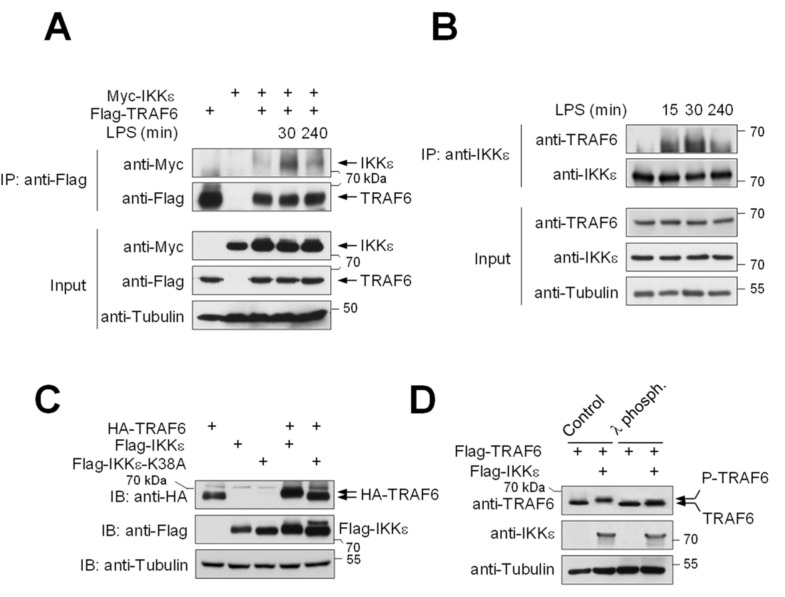
LPS-inducible interaction between IKKε and TRAF6. (**A**) HEK293 cells stably expressing TLR4 (293 TLR4) were transfected to express Myc-IKKε either alone or along with Flag-TRAF6. Cells were treated with LPS (2 µg/mL) for the indicated periods and lysed under native conditions. One aliquot of the extract was used for the input control; the remaining material was used for immunoprecipitation with anti-Flag antibodies. After elution of bound proteins, coprecipitated IKKε was visualized by Western blotting with anti-Myc antibodies. (**B**) HEK293 TLR4 cells were stimulated with LPS, as shown, and one aliquot of each cell extract was used for co-immunoprecipitation using anti-IKKε antibodies. (**C**) HEK 293 cells were transfected to express HA-TRAF6 alone or together with Flag-IKKε or Flag-IKKε-K38A, as shown. Cells were lysed under denaturing conditions, TRAF6 and the slower migrating form were detected by Western blotting using anti-HA antibodies. (**D**) HEK293 cells transfected to express Flag-TRAF6, alone or together with Flag-IKKε, were lysed under native conditions and incubated with λ-phosphatase for 20 min. TRAF6 and its phosphorylated form were detected by Western blotting with anti-TRAF6 antibodies.

We had previously found the regulation of IKKε by SUMOylation [41] and were interested in learning more about the regulation of this kinase. During our experiments, we made the serendipitous observation that IKKε and TRAF6 showed a weak interaction in untreated cells (Figure 1A), which is consistent with the published literature [42]. However, the interaction between IKKε and TRAF6 was significantly enhanced after LPS stimulation, as observed for epitope-tagged proteins (Figure 1A) and the endogenous proteins (Figure 1B). In the course of these experiments, we noted that co-expression of IKKε led to the occurrence of a slower migrating TRAF6 band in the presence of co-expressed IKKε, but not in cells expressing the kinase inactive IKKε-K38A variant (Figure 1C). To test whether this upshift is due to TRAF6 phosphorylation, HEK293 cells transfected to express Flag-TRAF6 alone or together with Flag-IKKε were lysed under native conditions and incubated with λ-phosphatase. Immunoblotting showed that treatment with λ-phosphatase reverted the IKKε-triggered upshift, suggesting that the slower migrating band corresponds to phosphorylated TRAF6 (Figure 1D).

**Figure 2:**
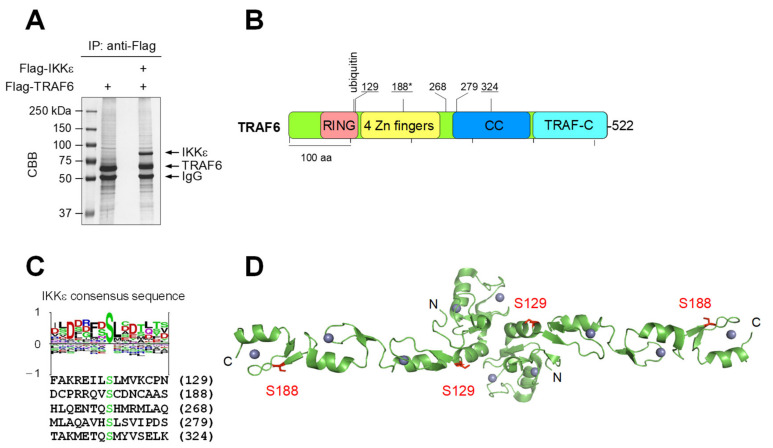
Mapping of IKKε-mediated TRAF6 phosphorylation sites. (**A**) HEK293T cells were used to express Flag-tagged forms of IKKε and TRAF6, as shown. Native extracts were produced and Flag-tagged proteins were purified by immunoprecipitation using anti Flag antibodies. One aliquot of the immunoprecipitated material was analyzed by SDS-PAGE and Coomassie brilliant blue (CBB) staining; the positions of molecular weight markers are indicated. The remaining material was used for SDS-PAGE, followed by excision of TRAF6 and mass spectrometric identification of TRAF6 phosphorylation sites. (**B**) Domain organization of TRAF6: RING = Really Interesting New Gene, Zn finger = zinc finger, CC = coiled coil region, TRAF-C = conserved C-terminal TRAF domain. The position of the phosphorylated serines identified via mass spectrometry are shown, the asterisk (*) indicates a known phosphorylation site, and the underlined residues are conserved in mouse TRAF6. (**C**) The sequences around the identified modification sites are listed and compared to the IKKε consensus phosphorylation sequence logo (deduced from 106 different input sequences) from the PhosphoSitePlus^®^ database. (**D**) The positions of TRAF6 phosphorylation sites on regions with available structural information are shown. The crystal structure showing the dimeric N-terminal domains of TRAF6 representing amino acids 54–210 (PDB code: 3HCS) was used to highlight the positions of two phosphorylated amino acids in red using Pymol.

To identify the IKKε-mediated phosphorylation sites, a mass spectrometry approach was used. Flag-tagged TRAF6 was expressed either alone or together with Flag-IKKε in HEK293T cells, followed by immunoprecipitation of both Flag-tagged proteins and mass spectrometry (Figure 2A and Figure S1). These experiments allowed the identification of IKKε-dependent phosphorylation at five serines at positions 129, 188, 268, 279 and 324, as schematically shown in Figure 2B. Constitutive phosphorylation at Ser 188 has already been identified [43], while all other phosphorylation sites have not yet been reported. The sequences surrounding the modification sites match well to a IKKε consensus motif (Figure 2C). Visualization of the phosphorylation sites on the TRAF6 part with available structural information shows the positions of Ser 129 and Ser 188 on the N-terminal dimerization and Ubc13 interaction domain (Figure 2D). The position of Ser 129 at the end of the α-2 Helix is located within a region that has been shown to interact with the donor ubiquitin [44].

### 3.2. TRAF6 Phosphorylation Counteracts Constitutive and LPS-Inducible Decay

**Figure 3:**
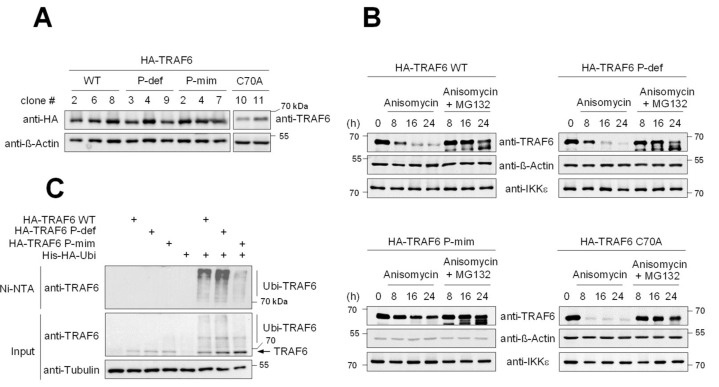
Generation and characterization of cell lines expressing mutant TRAF6. (**A**) TRAF6-deficient cells were transfected to stably express the indicated TRAF proteins that were C-terminally fused to a tandem HA-tag; the clone numbers are indicated on the top. (**B**) The indicated cell lines were incubated with Anisomycin (7.5 µg/mL), either alone or in combination with MG132 (20 µM), for the indicated periods. Cell extracts were prepared and analyzed for expression of the indicated proteins by Western blotting. (**C**) The indicated cell lines were used to express His-tagged ubiquitin, as shown. Cells were lysed under denaturing conditions, one aliquot was used for the input sample, while the remaining material was used to enrich His6-ubiquitinated proteins on Ni-NTA beads. The eluted samples were analyzed by Western blotting to detect TRAF6 ubiquitination using anti-TRAF6 antibodies.

To address the functional relevance of these phosphorylation events in vivo, we aimed to reconstitute the expression of wildtype (WT) and mutated forms of TRAF6 in TRAF6-deficient cells. To do so, TRAF6 was knocked-out using the CRISPR-Cas9 system in 293 TLR4 cells (Figure S2A,B). These TRAF6-deficient cells were used to stably reconstitute the expression of tandem HA-tagged forms of various TRAF6 variants: the WT form, phosphorylation-deficient TRAF6 where all the phosphorylated amino acids identified in this study were mutated to alanine (TRAF6 P-def), phospho-mimetic TRAF6 with Ser to Glu mutations in all phosphorylation sites identified here (TRAF6 P-mim), and, as a further control, the catalytically inactive TRAF6 C70A zinc finger mutant. Cells expressing similar levels of these TRAF6 variants comparable to the level of endogenous TRAF6 were generated (Figure 3A) and analyzed further. As TRAF6 stability is regulated in response to many external cues including virus infections, micro RNAs and inflammation [11,12,13], it was interesting to compare the protein stabilities of the different TRAF6 forms. Cells were incubated for different periods with the protein synthesis inhibitor Anisomycin and protein extracts were prepared to determine TRAF6 protein amounts. While the expression of TRAF6 WT and TRAF6 P-def was significantly reduced 8 h after translation inhibition, marked differences were observed for TRAF6 C70A and TRAF6 P-mim (Figure 3B). While TRAF6 C70A was highly instable and almost undetectable after 8 h of Anisomycin treatment, the TRAF6 P-mim mutant showed only very minor protein decay. TRAF6 degradation did not occur in the presence of the proteasome inhibitor MG132 (Figure 3B), consistent with the notion that basal TRAF6 decay is mediated by the ubiquitin/proteasome system. To test the impact of TRAF6 phosphorylation on its basal ubiquitination, the different cell lines expressing TRAF6 variants were transfected to express His6-tagged ubiquitin, followed by the enrichment of ubiquitinated proteins on Ni-NTA columns. This experiment revealed significantly impaired basal autoubiquitination of TRAF6 P-mim (Figure 3C).

**Figure 4:**
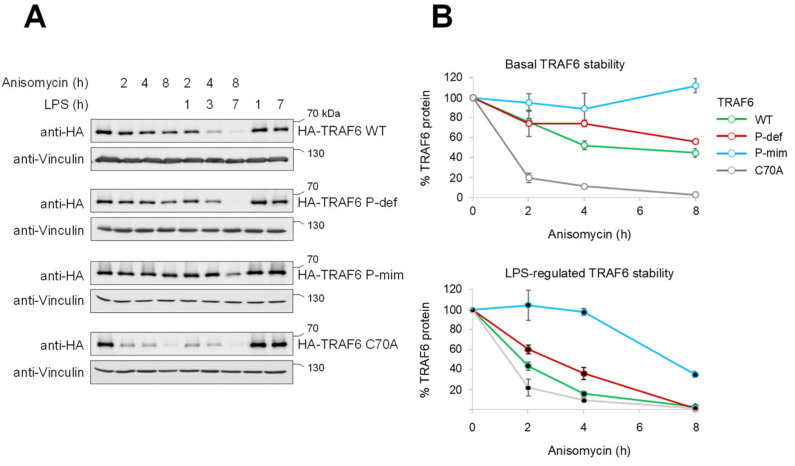
Analysis of LPS/Anisomycin-dependent TRAF6 degradation. (**A**) The indicated cell lines were incubated with Anisomycin either alone or in combination with LPS for the indicated periods. Cell extracts were prepared and analyzed for expression of the proteins, as shown. (**B**) Triplicates of the Western blots displayed in (**A**) were used for protein quantification using the ChemiDoc Imaging System. Relative protein amounts were normalized to Actin levels, and TRAF6 expression in untreated cells was arbitrarily set to 100%; the error bars show standard deviations.

It was then interesting to investigate whether TRAF6 phosphorylation also affects its stability in response to cell stimulation. To address this question, cells were stimulated for different periods with LPS, in the absence or presence of Anisomycin, to prevent re-synthesis of the eliminated protein and to enable proper detection of changes in TRAF6 levels. Western blot experiments revealed that LPS alone did not result in reduced TRAF6 levels (most likely due to the re-synthesis of TRAF6, as the effects of LPS were only visible in the presence of Anisomycin), and LPS-triggered TRAF6 decay was decreased in TRAF6 P-mim cells (Figure 4A). A quantitative analysis of basal and LPS-induced TRAF6 decay, showing the stabilization of TRAF6 P-mim and also the destabilization of TRAF6 C70A, is displayed in Figure 4B.

### 3.3. TRAF6 Phosphorylation Counteracts TRAF6 Elimination by Autophagy

**Figure 5:**
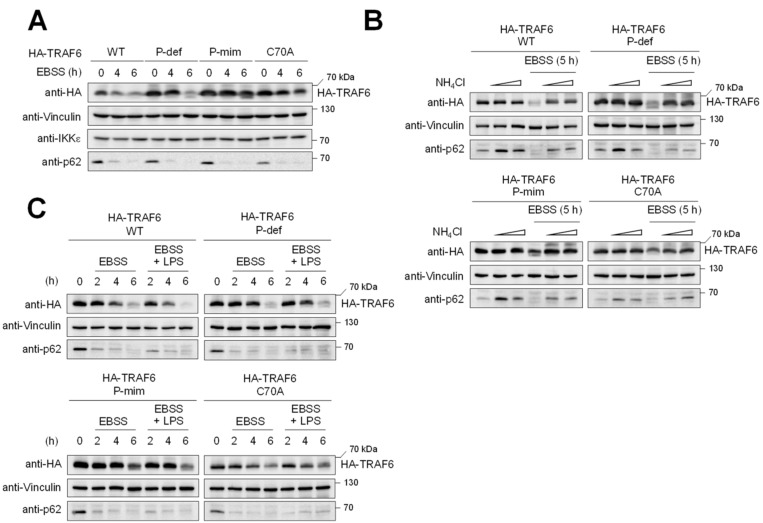
Analysis of TRAF6 degradation during autophagy. (**A**) The indicated cell lines were incubated in autophagy-inducing EBSS medium for the indicated periods. Cell extracts were analyzed by immunoblotting to visualize the decay of TRAF6 and of p62, a well-known substrate of the autophagosome. (**B**) Cells were incubated in the absence or presence of EBSS medium and/or NH_4_Cl (10–50 mM), as shown. The effects on the amount of TRAF6 and the other indicated proteins were measured by immunoblotting. (**C**) The experiment was conducted as in (**B**) with the exception that cells were incubated with EBSS and/or LPS.

As TRAF6 is involved in the regulation of diverse biological processes, it was interesting to study whether phosphorylation-dependent control of TRAF6 stability also occurs in response to further cues. To investigate the impact of autophagy on TRAF6, cells expressing the different variants of this ubiquitin E3 ligase were incubated in EBSS (Earle’s balanced salts solution) medium, which represents poor nutrient conditions [45]. This treatment caused the induction of autophagy (indicated by p62 degradation) and led to reduced levels of TRAF6 WT in 293 TLR4 cells (Figure 5A). Similar results were obtained in further cell systems such as HeLa cells (Figure S3). While the amounts of TRAF6 WT and TRAF6 P-def were clearly diminished 6 h post induction of autophagy, the phospho-mimetic TRAF6 mutant remained largely stable under these conditions (Figure 5A). Interestingly, the IKKε expression level did not change upon starvation. This result suggests that TRAF6 phosphorylation blocks TRAF6 decay also in autophagic cells. In this setting, TRAF6 degradation proceeds by autophagy and, accordingly, it was largely abrogated by the lysosomal inhibitor NH_4_Cl (Figure 5B). In contrast, it was only incompletely rescued by a proteasome inhibitor (Figure S4), most probably due to the effects on ongoing basal degradation. Interestingly, the TRAF6 C70A mutant that displayed an increased decay in LPS/Anisomycin-treated cells (see Figure 4A,B) was stabilized in cells undergoing autophagy (Figure 5A), suggesting the occurrence of different degradation pathways. As autophagy can also be induced by inflammatory signals such as LPS [21,46], we investigated whether LPS and EBSS cooperate in TRAF6 degradation during autophagy. The simultaneous induction of autophagy by LPS together with EBSS resulted in an increased TRAF6 decay when compared to EBSS treatment alone (Figure 5C). In addition, in this experimental setting, the phospho-mimetic TRAF6 variant and the TRAF6 C70A mutant showed increased stability. In EBSS-treated cells, the decay of TRAF6 occurred significantly later when compared to p62, suggesting that TRAF6 is eliminated rather late during autophagy.

### 3.4. The E3 Ligase Function of TRAF6 and Its Phosphorylation Differentially Shape LPS-Triggered Signaling Networks

**Figure 6:**
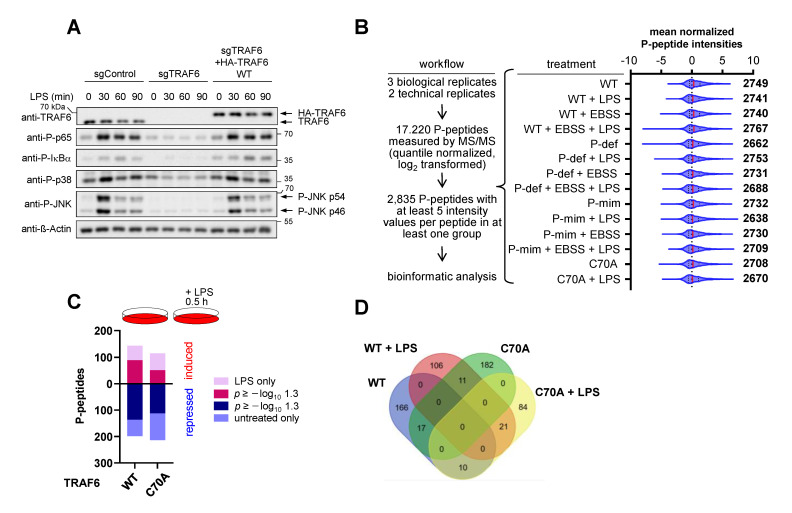
Analysis of LPS-induced TRAF6-dependent phosphorylation networks. (**A**) The indicated cells, either expressing endogenous TRAF6, deleted in TRAF6 or reconstituted to express TRAF6 in fusion with a C-terminal tandem HA tag, were stimulated for various time periods with LPS. Cell extracts were analyzed for basal and LPS-inducible phosphorylation of signaling proteins with specific antibodies, as shown. Note that the tandem HA tag increases the molecular weight of TRAF6. (**B**) Schematic display of the experimental setup and cell treatment. EBSS treatment was always performed for 5 h and LPS treatment for 30 min. Cells experiencing consecutive EBSS and LPS treatment were allowed to recover for 30 min following starvation. The subsequent LC-MS/MS workflow is indicated. The Violin plots show total numbers and mean normalized phosphopeptide intensities under the various conditions. (**C**) The top shows the experimental design; the lower part shows the number of phosphopeptides that were increased or decreased after LPS stimulation in TRAF6 WT or TRAF6 C70A expressing cells. We separately show regulated peptides where a *p*-value could not be calculated due to the fact that phosphorylation was not occurring/detected in at least one of the samples. (**D**) The Venn diagram shows a comparison of phosphopeptides (up- or downregulation) determined in unstimulated or LPS-treated cells expressing TRAF6 WT or TRAF6 C70A.

Although lacking any intrinsic kinase activity, the TRAF6 protein is necessary to trigger LPS-inducible activation of downstream protein kinases including JNK (c-Jun N-terminal kinase) and IκB kinases (IKKs) (Figure 6A). Given the relevance of TRAF6 as a central mediator of immunity and cell homeostasis [17,47,48,49], we were interested in analyzing the impact of TRAF6 phosphorylation on downstream signaling networks. TRAF6-dependent signaling causes activation of protein kinase networks that were interrogated by phosphoproteomic analyses to address the following questions: (I) What is the relative contribution of TRAF6 phosphorylation and its E3 ligase function to the regulation of basal and stimulus-induced phosphorylation networks? (II) What is the impact of autophagy on the ability of TRAF6 and its phospho-mutants to mediate subsequent LPS-regulated phosphorylation changes? To address these questions, cells expressing TRAF6, and mutants thereof, were exposed to nutrient poor medium and/or LPS in various combinations, as schematically shown in Figure 6B. Cell extracts from three biological replicates were digested with trypsin and phosphorylated peptides were enriched via immobilized metal affinity chromatography (IMAC) columns, followed by MS/MS analysis in two technical replicates. This allowed the identification of 2835 peptides with at least five intensity values per peptide in at least one group. A display of mean normalized phospho-peptide intensities ensured the comparability of data sets (Figure 6B and Figure S5). For the subsequent analyses, we display results from phosphopeptides where a *p*-value could be calculated, but also from samples without a *p*-value, due to the fact that a phosphorylation was not occurring/detected under at least one condition, such as in untreated cells. All data from the phosphoproteomic analysis are displayed in Table S2. In cells expressing WT TRAF6, the treatment with LPS caused increased phosphorylation of 142 peptides and decreased phosphorylation of 198 phosphopeptides. The dynamic range was fully maintained in cells expressing TRAF6 C70A (Figure 6C). However, a comparison of the phosphopeptides revealed limited overlap between phosphopeptides co-regulated by TRAF6 and its E3 ligase deficient mutant (Figure 6D).

**Figure 7:**
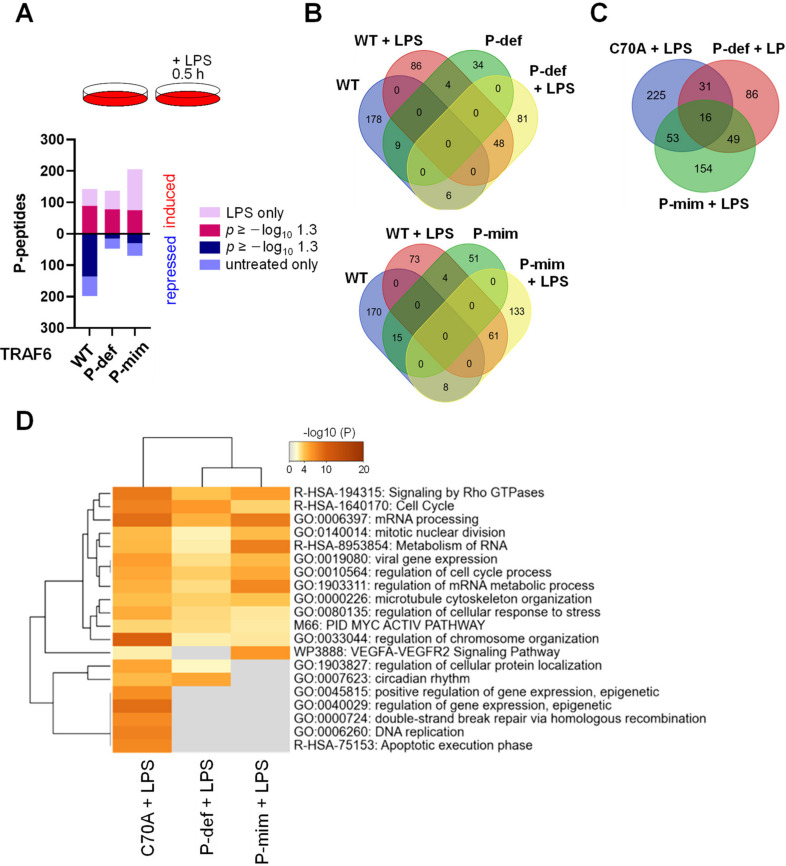
The impact of TRAF6 phosphorylation on LPS-triggered phosphorylation networks. (**A**) The top shows the experimental design; the lower part shows the number of phosphopeptides that were increased or decreased after LPS stimulation in the cells expressing TRAF6 and its phospho-mutants. (**B**) The Venn diagram shows a comparison of phosphopeptides (up or downregulation) determined the in unstimulated or LPS-treated cells as indicated. (**C**) Venn diagram comparing the dynamically regulated phosphopeptides in cells expressing TRAF6 C70A or the indicated TRAF6 phosphorylation site mutants. (**D**). Protein IDs mapping to the unique groups of deregulated phosphopeptides shown in the Venn diagram of (**C**) were further used to perform an overrepresentation (ORA) analysis using Metascape. The enriched pathway terms are indicated; the grey fields visualize lack of enrichment.

These results suggest that (I) the ubiquitin conjugating function of TRAF6 is critically involved in the shaping of LPS-induced phosphorylation networks, and (II) the signaling function of TRAF6 does not entirely rely on its E3 ligase activity, likely by functioning as a scaffold that allows dimerization with further TRAFs and signaling proteins. The comparative analysis of TRAF6 phosphorylation on its ability to trigger downstream signaling networks revealed an intact ability of the TRAF6 phospho-mutants to trigger LPS-induced phosphorylations, while the number of downregulated phosphopeptides was markedly reduced (Figure 7A). In addition, the phosphorylation-defective or phospho-mimetic TRAF6 forms showed strong changes between the phosphopeptide patterns in untreated and LPS-stimulated cells, when compared to the TRAF6 WT protein (Figure 7B). Given that the E3 ligase function of TRAF6 and its phosphorylation shape differential signaling outputs, it was interesting to analyze those further. The three TRAF6 mutants showed only a limited overlap in substrates (Figure 7C), suggesting that phosphorylation critically defines the signaling output. Although TRAF6 modification drastically altered the regulated phosphopeptides, mapping of the differentially regulated phosphopeptides to GO/KEGG pathways using Metascape software revealed that the phosphorylation targets still fall in largely similar functional categories covering processes such as regulation of mRNA processing, cell cycle and the stress response (Figure 7D). Cells expressing the TRAF6 phospho-mutants had lost dynamic phosphorylation of proteins involved in some processes including the regulation of apoptosis, DNA replication and gene expression (Figure 7D and Figure S6), suggesting that modification of TRAF6 reshapes the signaling output.

### 3.5. TRAF6-Dependent Signaling Networks Are Regulated by Nutrient Availability

**Figure 8:**
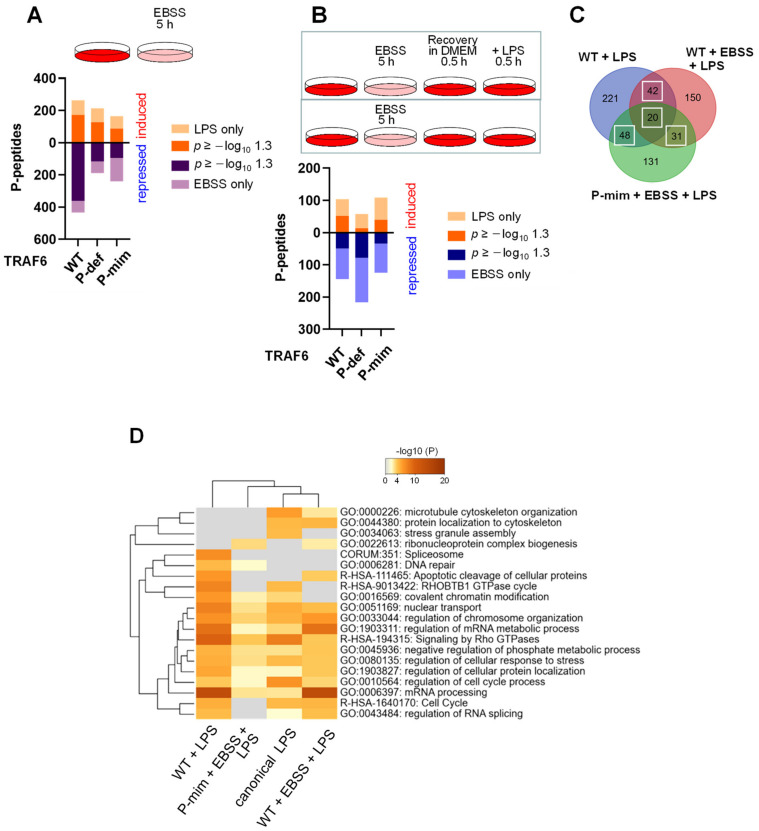
The impact of autophagy and TRAF6 phosphorylation on LPS-triggered phosphorylation networks. (**A**) The top shows the experimental design, the lower part shows the number of phosphopeptides that were increased or decreased after EBSS treatment in the cells expressing TRAF6 and its phospho-mutants. (**B**) Cells were exposed to EBSS, followed by re-addition of complete medium. Then, cells remained untreated (controls) or were stimulated with LPS, as shown, followed by phosphoproteomic analysis. The number of regulated phosphopeptides in the various cell lines is shown. (**C**) Venn diagram comparing the dynamically regulated phosphopeptides from LPS-induced cells in dependence on starvation and TRAF6 phosphorylation as shown. The white boxes mark commonly regulated phosphopetides that are defined as canonical LPS-regulated. (**D**) Protein IDs that map to the canonical group of LPS-regulated phophopeptides as well as to the unique phosphopeptides shown in the Venn of (**C**) were subjected to ORA analysis.

To test the impact of autophagy and basal TRAF6 phosphorylation on signaling cascades, cells expressing TRAF6 WT, TRAF6 P-def or TRAF6 P-mim were left untreated or exposed for 5 h to EBSS medium, followed by the analysis of the phosphorylation status. In cells expressing TRAF6 WT, the induction of autophagy resulted in dynamically regulated changes in the phosphoproteome with a bias towards reduced phosphorylation (Figure 8A). In cells expressing TRAF6 phospho-mutants, the dynamic range of autophagy-regulated phosphopeptides was diminished (Figure 8A). We then investigated the functional consequences of autophagy on the ability of cells to trigger a subsequent signaling response to LPS. To address this question, cells were starved for 5 h in EBSS medium, followed by the addition of DMEM (30 min) to allow cell recovery, and LPS stimulation for another 30 min. A phosphoproteomic analysis showed an increased downregulation of LPS-regulated phosphorylations in cells expressing a phosphorylation-deficient TRAF6 mutant (Figure 8B). A comparative analysis of LPS-regulated phosphopeptides showed that prior induction of autophagy led to a reduced number of LPS-regulated phosphorylations. There was a core of 141 phosphopeptides that were dynamically regulated under all conditions in response to LPS treatment, here referred to as canonical phosphopeptides (Figure 8C). In addition, variable numbers of LPS-regulated phosphopeptides only occurred in a manner that was dependent on TRAF6 phosphorylation, or in cells that had experienced autophagy (Figure 8C). Mapping of the phosphopeptides to GO/KEGG pathways showed that the diversity and differences at the level of phosphopeptides is only partially reflected at the level of biological processes, which show significant overlap (Figure 8D). The previous experience of autophagy changed the phosphorylation patterns, leading to an underrepresentation of proteins involved in chromatin modification, DNA repair and stress granule assembly, while enabling phosphorylation of proteins involved in cytoskeleton organization. The EBSS/LPS-mediated processes were further affected by TRAF6 phosphorylation, leading to the underrepresentation of proteins that regulate RNA splicing, and reorganization of the cytoskeleton and the cell cycle (Figure 8D).

## 4. Discussion

**Figure 9:**
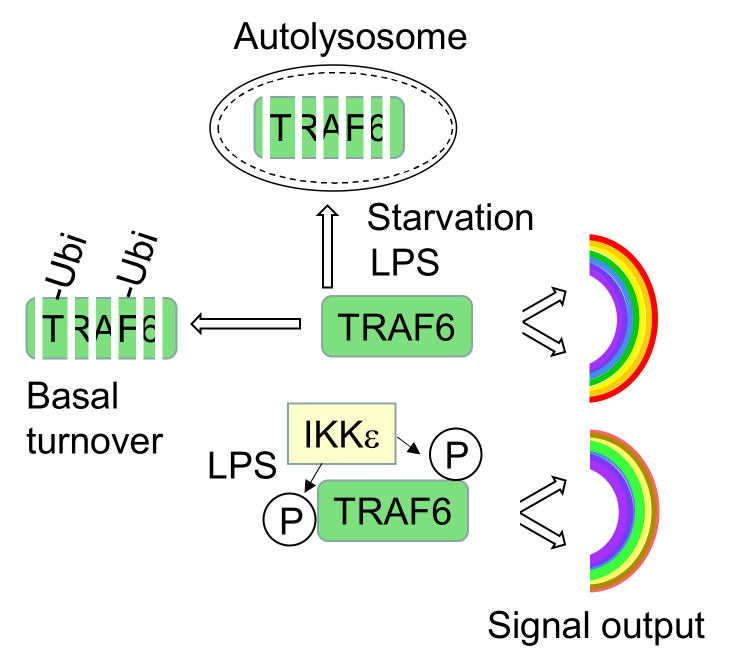
Schematic model summarizing the mechanisms and functions of TRAF6 phosphorylation for protein stability and downstream signaling networks.

The TRAF6 protein has been implicated in a large number of different biological processes, ranging from differentiation to inflammatory signaling and autophagy. This raises the need to specify the signaling output networks to suit the specific requirements of the biological response. This study shows that TRAF6 phosphorylation (I) affects the control of basal and autophagy-regulated TRAF6 abundance and (II) has a profound impact on downstream signaling networks, as schematically visualized in Figure 9.

Some LPS-induced signaling pathways such as processing and localization of RNA are not affected by TRAF6 phosphorylation, while other pathways including cytoskeletal organization as well as cell division and proliferation are shaped by TRAF6 modification (Supplementary Figure S6). All of these distinct downstream pathways can be associated with immune signaling, which goes along with immune cell proliferation and cytoskeletal reorganization [50], but may also affect further unrelated TRAF6-mediated biological processes.

The regulation of TRAF6 stability has been observed in a number of different settings [11,12,13]. Infection of cells with a number of various viruses including Hepatitis C virus (HCV), Coronaviruses (CoV) and the bovine herpes virus (BHV-1) leads to the degradation of TRAF6 to enable escape from host cell immune signaling by different decay pathways. The CoV-encoded protein ORF-9b instructs the E3 ligase AIP4 to trigger TRAF6 degradation by promoting K48-linked polyubiquitination and proteasomal degradation [51]. The same proteolytic pathway is employed by the BHV-1-encoded infected cell protein 0 (BICP0), which directly binds to TRAF6 in order to facilitate its ubiquitin/proteasome-mediated elimination [52]. In contrast, HCV infection leads to p62-dependent depletion of TRAF6 in autophagosomes [12]. In addition, dysregulated expression of cellular proteins, as it occurs in cancer, can lead to TRAF6 degradation. High expression levels of the kinases DRAK1 (death-associated protein kinase-related apoptosis-inducing kinase 1) or c-Cbl (Casitas B-lineage Lymphoma) lead to ubiquitin/proteasome-mediated TRAF6 decay [14,53].

This study shows that TRAF6 phosphorylation controls two distinct degradation pathways: (I) Basal and constitutive TRAF6 turnover occurs through the ubiquitin/proteasome system, and increased stability of phosphorylated TRAF6 could potentially be attributed to phosphorylation of Ser 129. This site is located in a region that mediates trans-interactions with the donor ubiquitin (see also Figure 2D). As it was shown that mutation of TRAF6 Ser 129 to Ala slightly decreased TRAF6 autoubiquitination [44], it is conceivable that the addition of a bulky and negatively charged phosphate group will have a more profound impact on TRAF6 autoubiquitination. Accordingly, we have observed reduced auto-ubiquitination of TRAF6 P-mim (see Figure 3C, but it remains to be seen whether the other phosphorylation sites also contribute to TRAF6 stabilization. (II) The second TRAF6 decay pathway occurs in response to metabolic perturbation and proceeds by autophagy. This study shows that autophagy-mediated TRAF6 elimination occurs rather late, as revealed by comparison to the decay kinetics of the early autophagy substrate p62. While TRAF6 participates in the induction phase of autophagy [20,21,54], its elimination in the late phase contributes to a reprogramming of inflammatory phosphorylation networks. We speculate that this might result in diminished inflammation, and accordingly anti-inflammatory effects of autophagy have also been suggested by the analysis of mice lacking the autophagy gene Atg16L1 (autophagy-related 16-like 1). These knockout animals have an increased host defense and are resistant to intestinal disease induced by the model bacterial pathogen *Citrobacter rodentium* [55]. The elevated immune response after elimination of autophagy genes is also seen in response to virus infections. Here, ablation of the Epg5 (ectopic P-granules autophagy protein 5 homolog) gene leads to increased baseline inflammation and resistance to lethal influenza virus infection [56].

We currently have no mechanistic explanation for the phosphorylation-dependent protection of TRAF6 from autophagic decay. Given the reduced ubiquitination of TRAF6 P-mim, one possible mechanism would involve the TRAF6 interactor p62, which serves as a receptor targeting ubiquitinated proteins to phagophores for degradation [57]. Alternatively, the addition of phosphate groups could affect protein/protein interactions or the conformation of TRAF6.

The function of TRAF6 phosphorylation in the specification of signaling output programs will not only be due to different expression levels, as discussed above, but probably also to effects on the strength and duration of protein/protein interactions, protein localization and enzymatic activities [58]. These mechanisms can create differentially composed multi-protein complexes that shape distinct signaling outputs. These interaction partners could be hetero-dimerization partners such as TRAF2, TRAF3 and TRAF5 [59], but also numerous other cellular proteins that bind in a constitutive or signal-regulated manner, as discovered here for IKKε. Basal or signal-induced TRAF6 phosphorylation can potentially affect the composition of ubiquitin chains on its target proteins, as TRAF6 may also form atypical ubiquitin linkages in addition to the known K63-branched chains [60]. Potential TRAF6-dependent downstream kinases feeding into these phosphorylation networks include TAK1 [61], the LPS-inducible TRAF6 interactor TBK1 [62], and probably also further kinases including IKKε. This might involve TRAF6-mediated ubiquitination of IKKε, which occurs in a manner that is dependent on the E3 ligase activity of TRAF6 its ability to dimerize (Figure S7). It will thus be interesting to study the functional consequences of TRAF6-mediated IKKε ubiquitination in the future.

## 5. Conclusions

This work identifies IKKε-mediated TRAF6 phosphorylation as a novel mechanism that controls TRAF6 stability and signal output. Phosphorylated TRAF6 is largely protected from basal ubiquitin/proteasome-mediated degradation and also from autophagy-mediated decay, thus providing another example that shows the cross-regulation between metabolic and inflammatory circuits. The metabolic regulation of TRAF6 stability and its implications for downstream signaling can probably also contribute to smoldering inflammation that is associated with aging and metabolic diseases such as diabetes and obesity.

## Data Availability

The data from the phosphoproteomic analysis are given in the Supplementary Table S2.

## Supplementary Materials

The following are available online at https://www.mdpi.com/article/10.3390/cancers13143618/s1, Figure S1: MS2 Spectra of TRAF6 phosphorylation sites only seen in presence of IKKε, Figure S2: (A) 293 TLR4 cells were transfected with the vector px459-TRAF6 which directs the synthesis of Cas9 and a sgRNA targeting the first exon of TRAF6. Transfected cells were selected by puromycin treatment and surviving clones were grown to colonies. A fraction of the indicated cell clones was lysed and equal amounts of protein were tested by immunoblotting for expression levels of TRAF6, Cas9 and Tubulin with specific antibodies. (B) TRAF6-deficient cells were tested for the occurrence of genetic changes by PCR and sequencing. The underlined areas were deleted and the G was inserted as shown, leading to frame-shifts and defect TRAF6 expression, Figure S3: HeLa cells were exposed for the indicated periods to EBSS medium and analyzed by Western blotting for expression of the indicated proteins, Figure S4: The indicated cell lines were incubated with EBSS medium for 5 h as shown and simultaneously treated with increasing amounts of the proteasome inhibitor Lactacystin (5, 10 µM), Figure S5: The Venn diagrams show the number of phosphopeptides measured under the indicated conditions in the various cells. The numbers in the brackets indicate (all P-peptides; unique P-peptides), Figure S6: Protein Ids mapping to the unique groups of deregulated phosphopeptides shown were further used to perform an overrepresentation (ORA) analysis using Metascape, Figure S7: HEK293 cells expressing Flag-IKKε alone or along with His6-ubiquitin and the indicated TRAF6 variants (C70A: defective in E3 ligase function; K124R: defective in autoubiquitination; R88A and R88A/F122A: defective in dimerization) were lysed under denaturing conditions, Figure S8: Whole Western Blots, Table S1: Antibodies and plasmids, Table S2: Phosphoproteomic analysis. References [63,64,65,66] are referred to in Supplementary Materials.
