# Supplementary material for: TRAF6 Phosphorylation Prevents Its Autophagic Degradation and Re-Shapes LPS-Triggered Signaling Networks
Source: Cancers (Basel). 2021 Jul 19;13(14):3618. doi: 10.3390/cancers13143618 (PMC8303406; doi:10.3390/cancers13143618)
Supplement: Supplementary file 1 [file cancers-13-03618-s001.zip › Supplementary Files.pdf]

Supplementary Materials

(A) TRAF6-S129

(R)EIL**pS**LMVK(C) (Mascot ion score: 29.02)

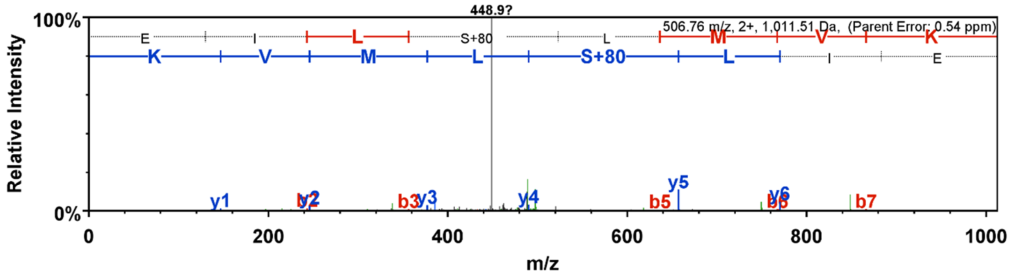

(B) TRAF6-S188

(R)RQV**pS**cDNcAASMAFEDK(E) (Mascot ion score: 34, missed cleavage)

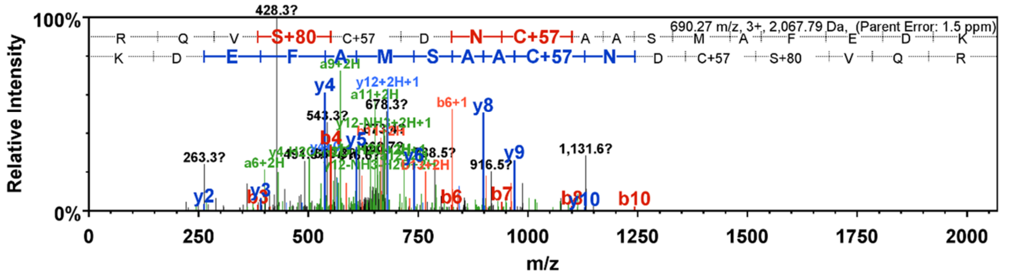

TRAF6-S188 (second evidence; MSA mode)

(R)QV**pS**cDNcAASmAFEDK(E) (Mascot ion score: 77.04)

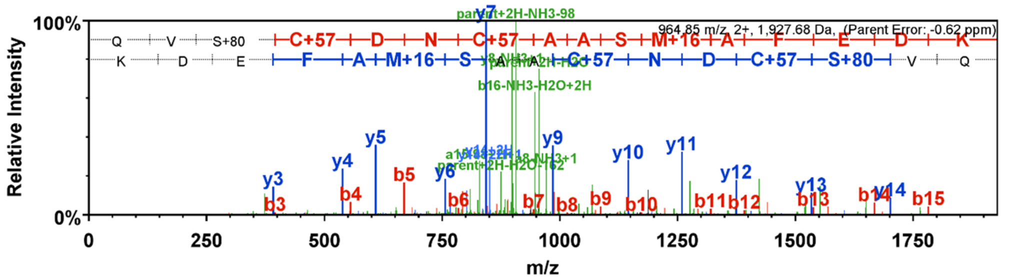

(C) TRAF6-S268

(R)HLQENTQ**pS**HMR(M) (Mascot ion score: 39)

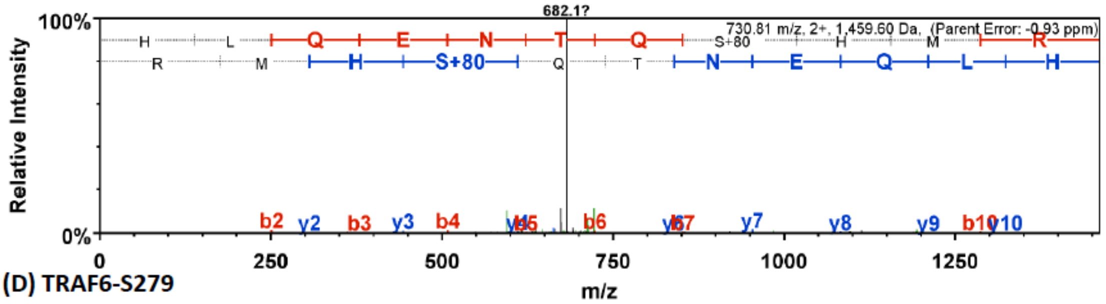

(D) TRAF6-S279

(R)MLAQAVH**pS**LSVIPDSGYISEVR(N) (Masot ion score: 74.46)

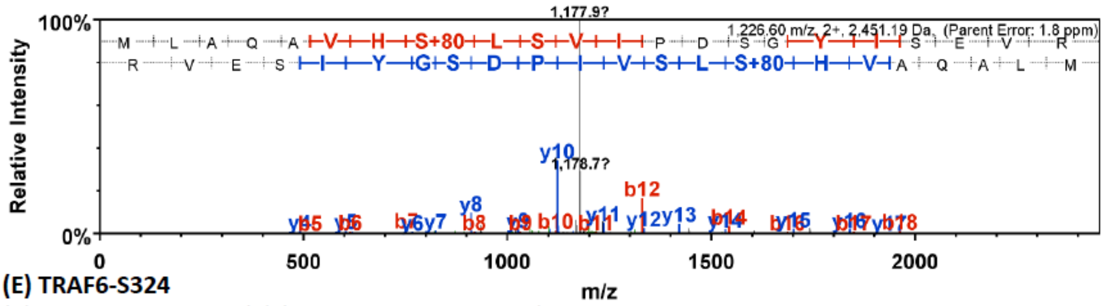

(E) TRAF6-S324

(K)METQ**pS**MYVSELKR(T) (Masot ion score: 74.46)

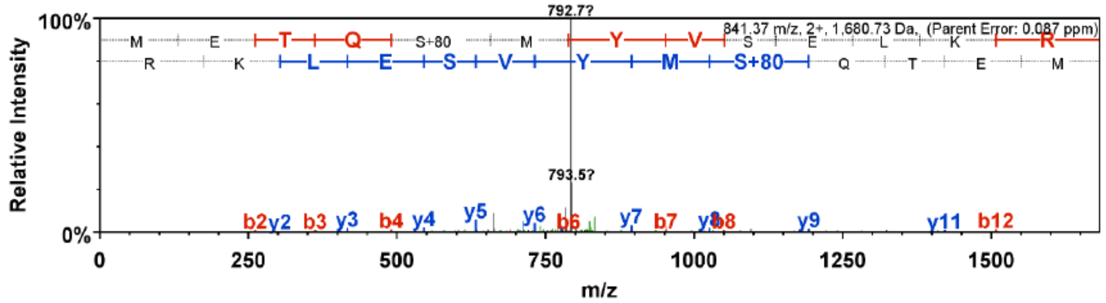

Figure S1. MS2 Spectra of TRAF6 phosphorylation sites only seen in presence of IKKε. Representative spectra for the sites **(A)** S129, **(B)** S188, **(C)** S268, **(D)** S279 and **(E)** S324 are shown. For S188 (B) and additional spectrum without missed cleavage and acquired by MSA (multi-stage activation) is shown. The phosphosites are shown in red.

A

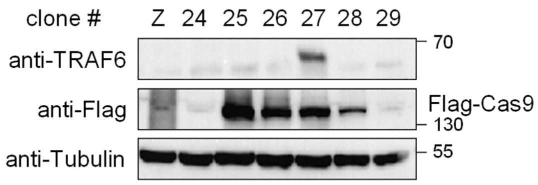

B

```
                                                +g
                                                 |
aca aaa gat gat agt gtg ggt gga act gcc agc acg ggg aac ctc tcc agc acg

 T   K   D   D   S   V   G   G   T   A   S   T   G   N   L   S   S   T

31
```

Figure S2. (A) 293 TLR4 cells were transfected with the vector px459-TRAF6 which directs the synthesis of Cas9 and a sgRNA targeting the first exon of TRAF6. Transfected cells were selected by puromycin treatment and surviving clones were grown to colonies. A fraction of the indicated cell clones was lysed and equal amounts of protein were tested by immunoblotting for expression levels of TRAF6, Cas9 and Tubulin with specific antibodies. **(B)** TRAF6-deficient cells were tested for the occurrence of genetic changes by PCR and sequencing. The underlined areas were deleted and the G was inserted as shown, leading to frame-shifts and defect TRAF6 expression.

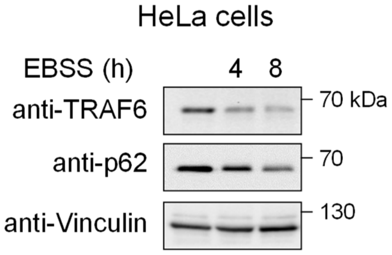

Figure S3. HeLa cells were exposed for the indicated periods to EBSS medium and analyzed by Western blotting for expression of the indicated proteins.

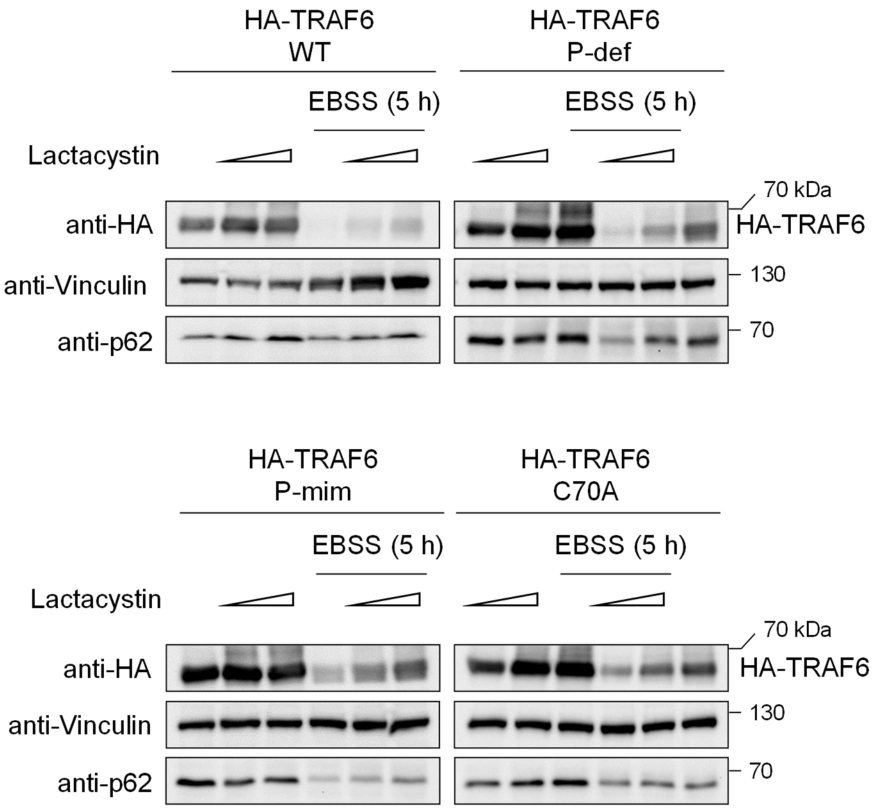

Figure S4. The indicated cell lines were incubated with EBSS medium for 5 h as shown and simultaneously treated with increasing amounts of the proteasome inhibitor Lactacystin (5, 10 μM). Cell extracts were prepared and analyzed for expression of the indicated proteins by Western blotting.

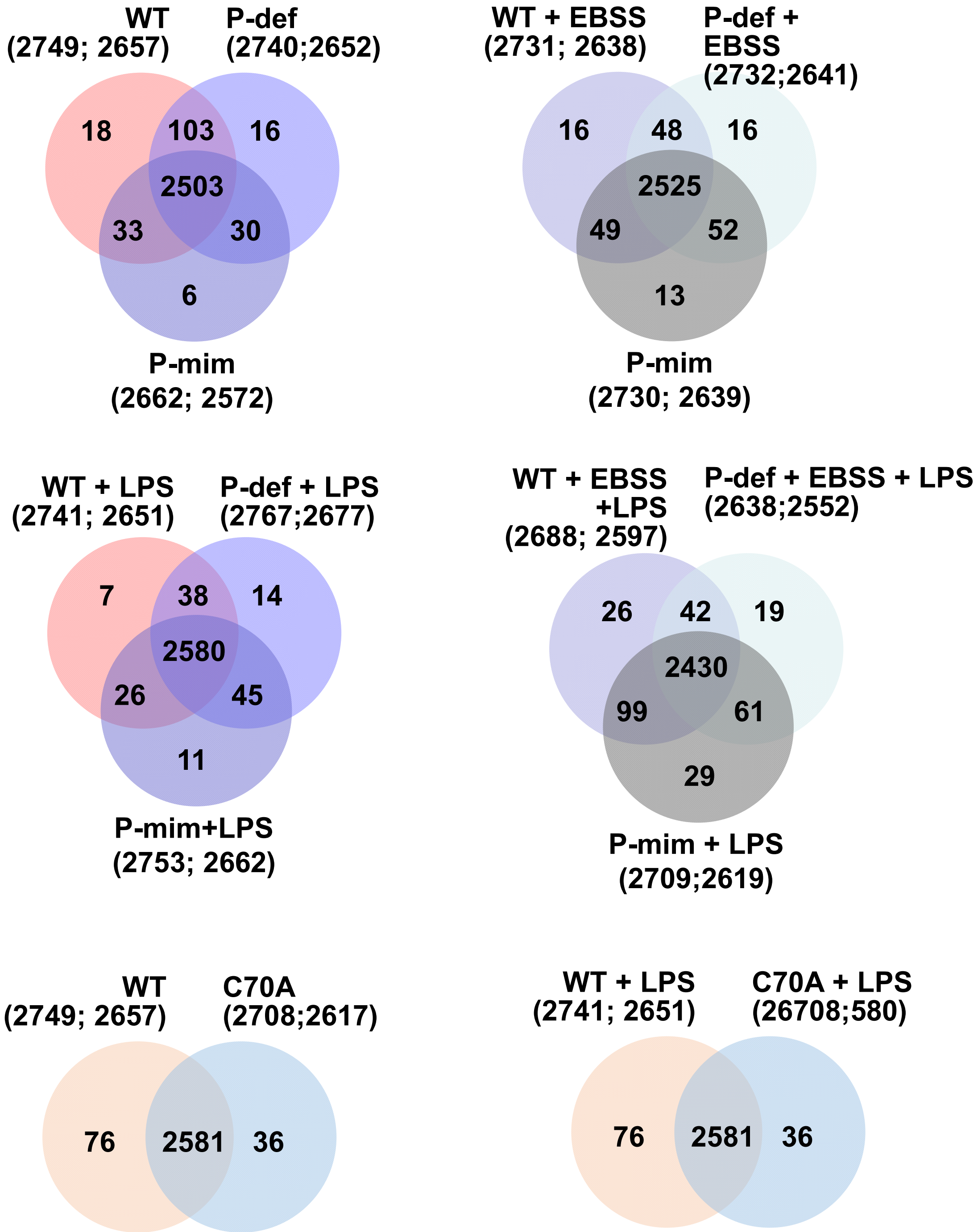

Figure S5. The Venn diagrams show the number of phosphopeptides measured under the indicated conditions in the various cells. The numbers in the brackets indicate (all P-peptides; unique P-peptides).

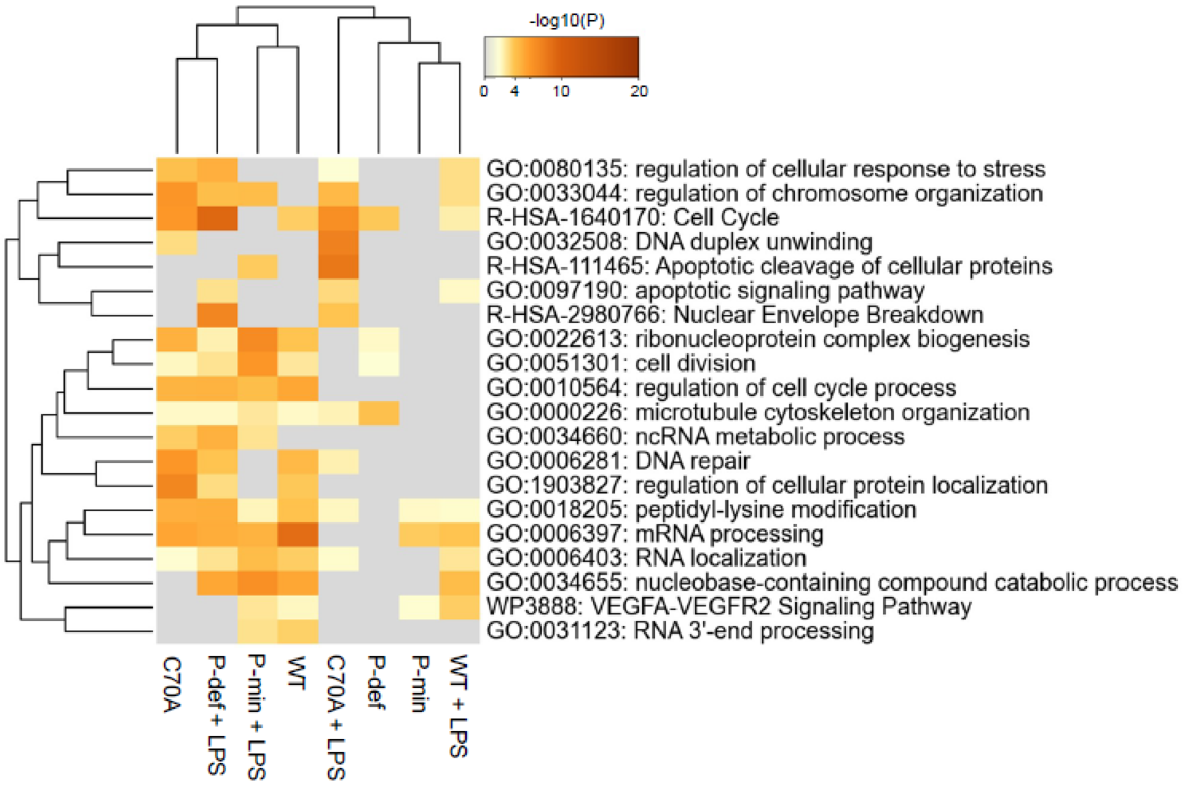

Figure S6. Protein Ids mapping to the unique groups of deregulated phosphopeptides shown were further used to perform an overrepresentation (ORA) analysis using Metascape. The enriched pathway terms are indicated, the grey fields visualize lack of enrichment.

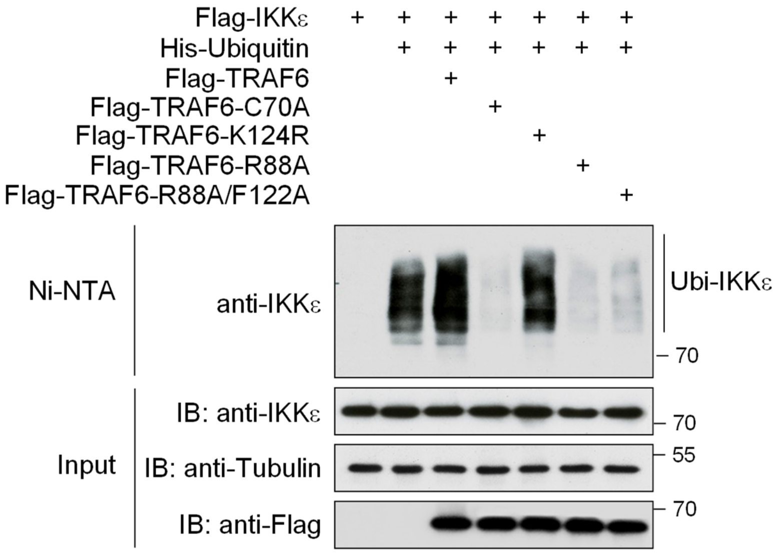

Figure S7. HEK293 cells expressing Flag-IKKε alone or along with His$_6$-ubiquitin and the indicated TRAF6 variants (C70A: defective in E3 ligase function; K124R: defective in autoubiquitination; R88A and R88A/F122A: defective in dimerization) were lysed under denaturing conditions. One aliquot was used for the input sample, while the remaining material was used to enrich His$_6$-ubiquitinated proteins on Ni-NTA beads. The eluted samples were analyzed by Western blotting to detect IKKε ubiquitination using anti-IKKε antibodies.

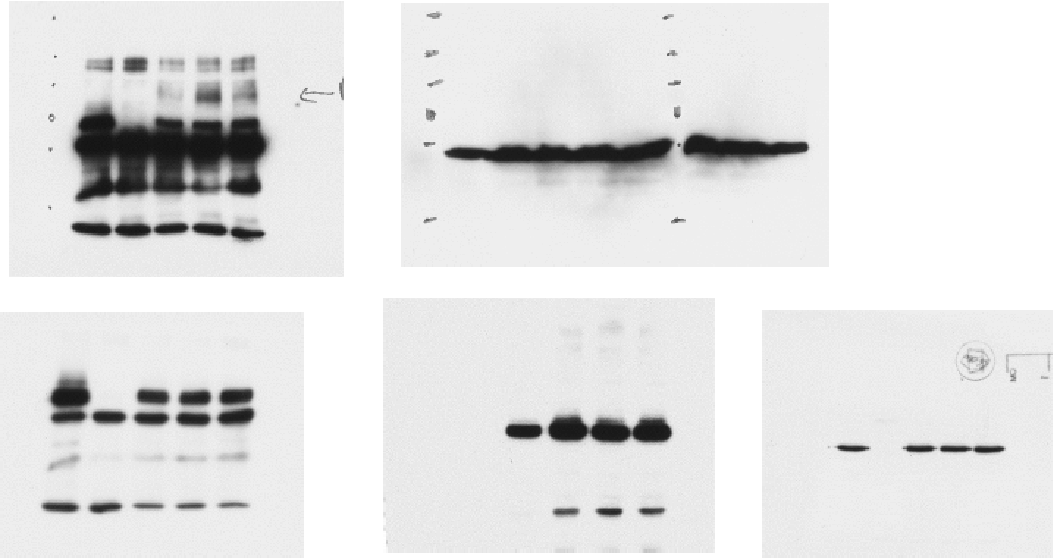

1A

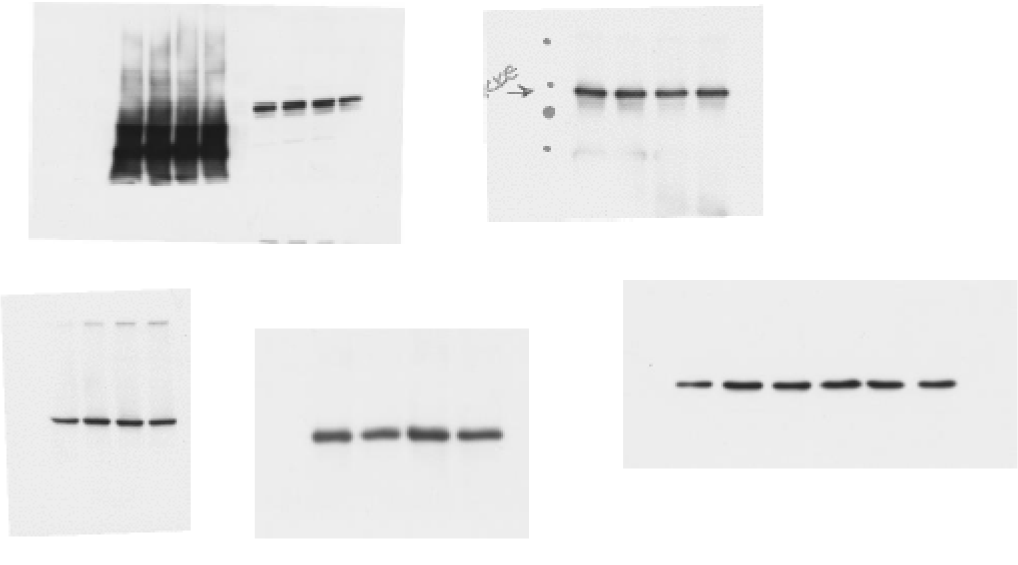

1B

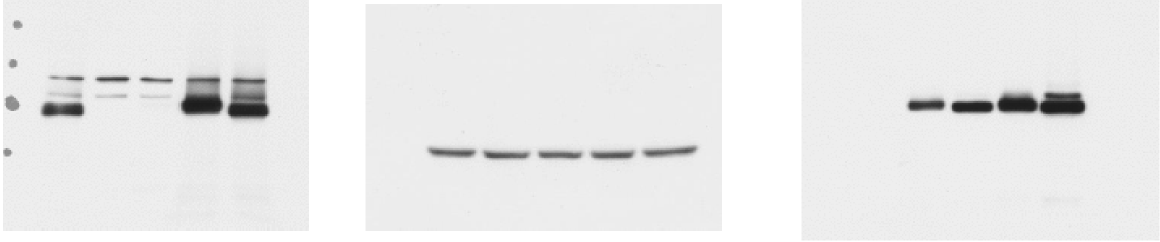

1C

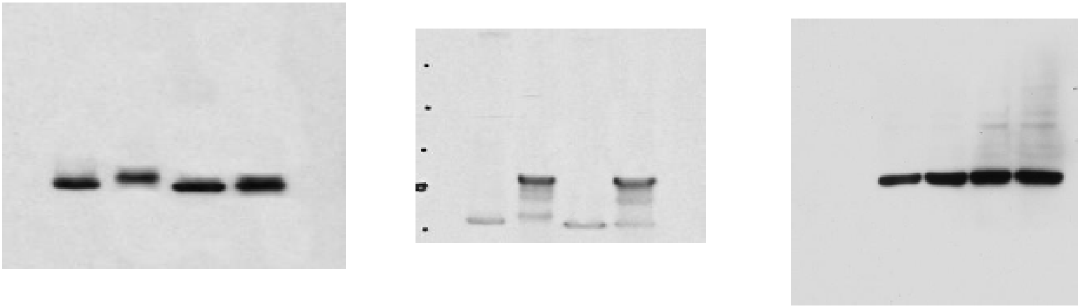

1D

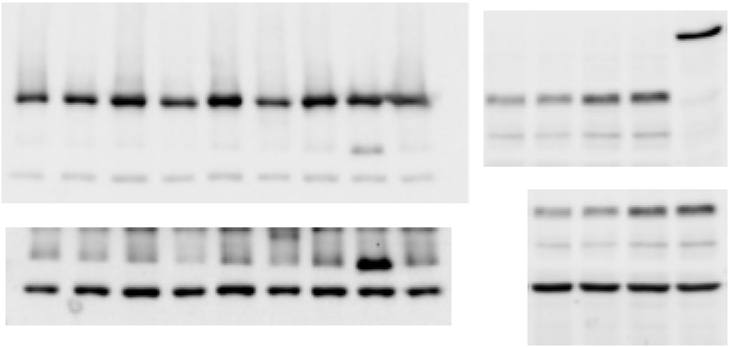

3A

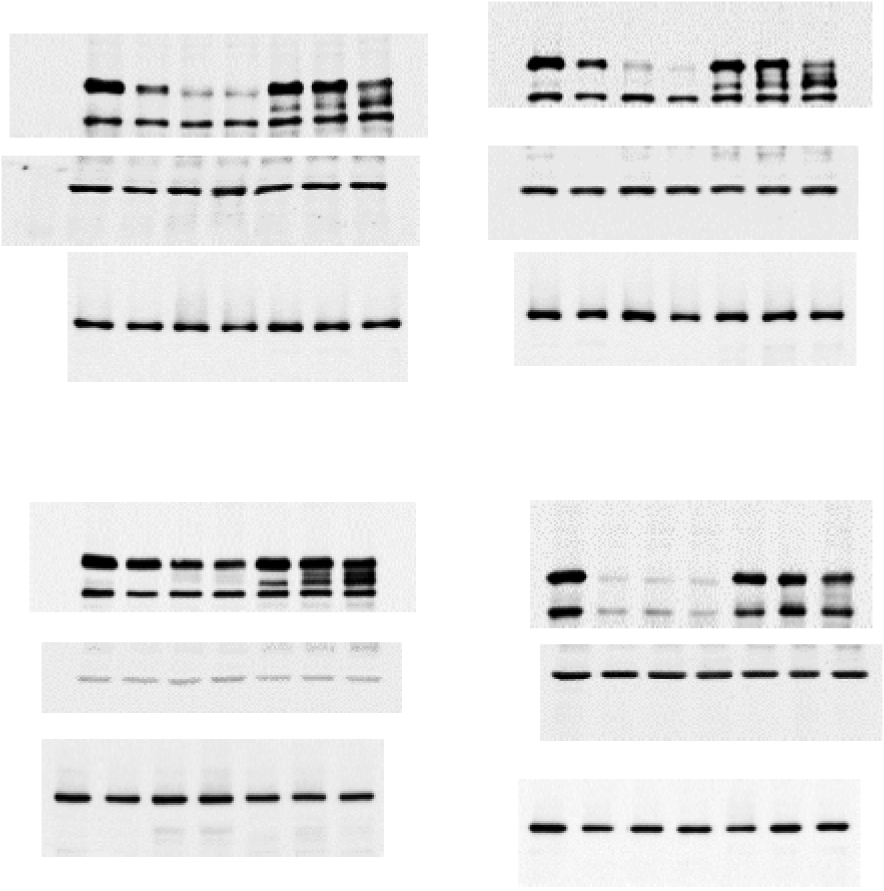

3B

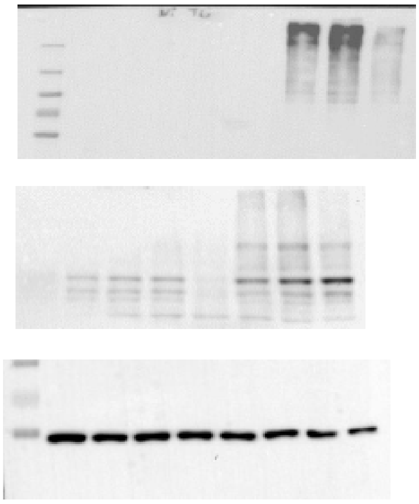

3C

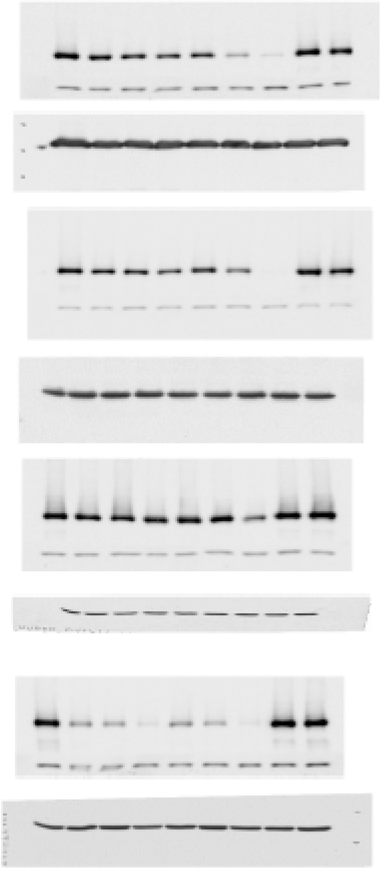

4A

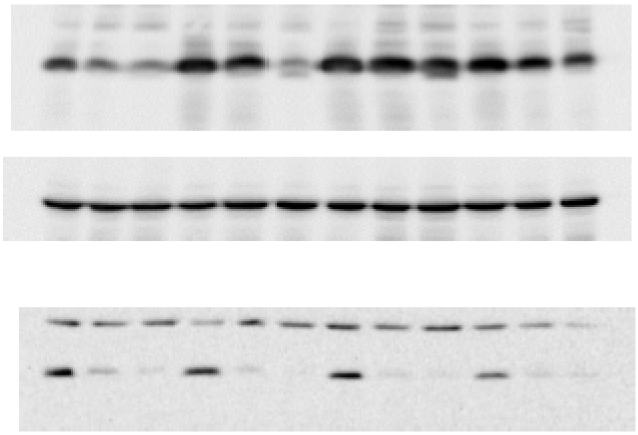

5A

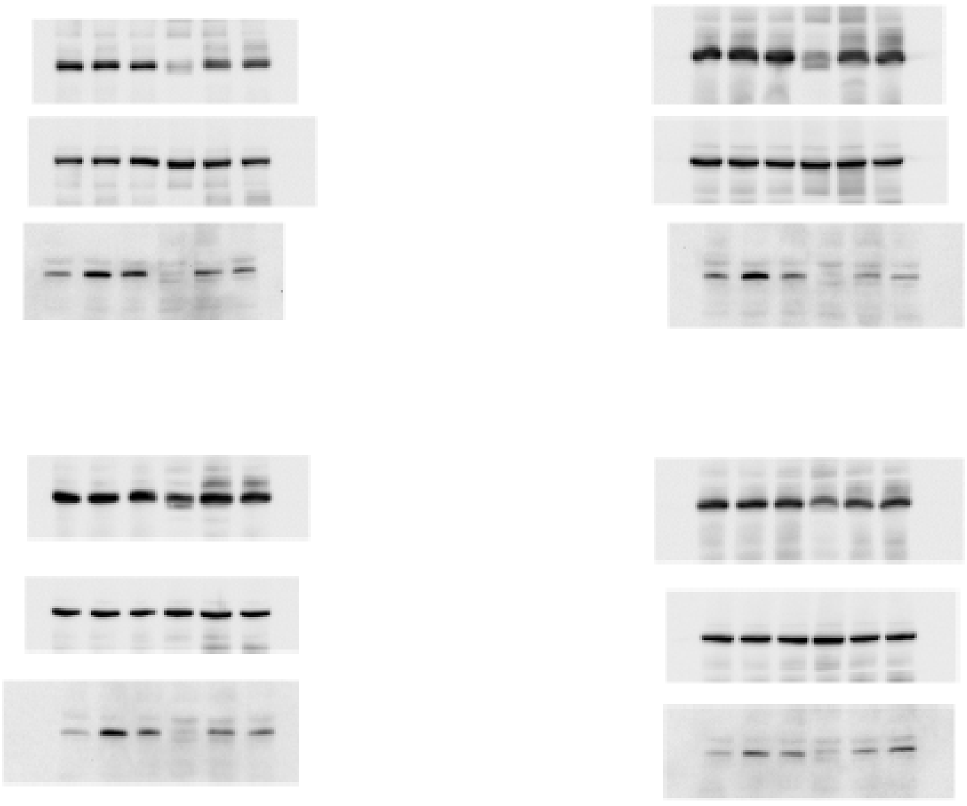

5B

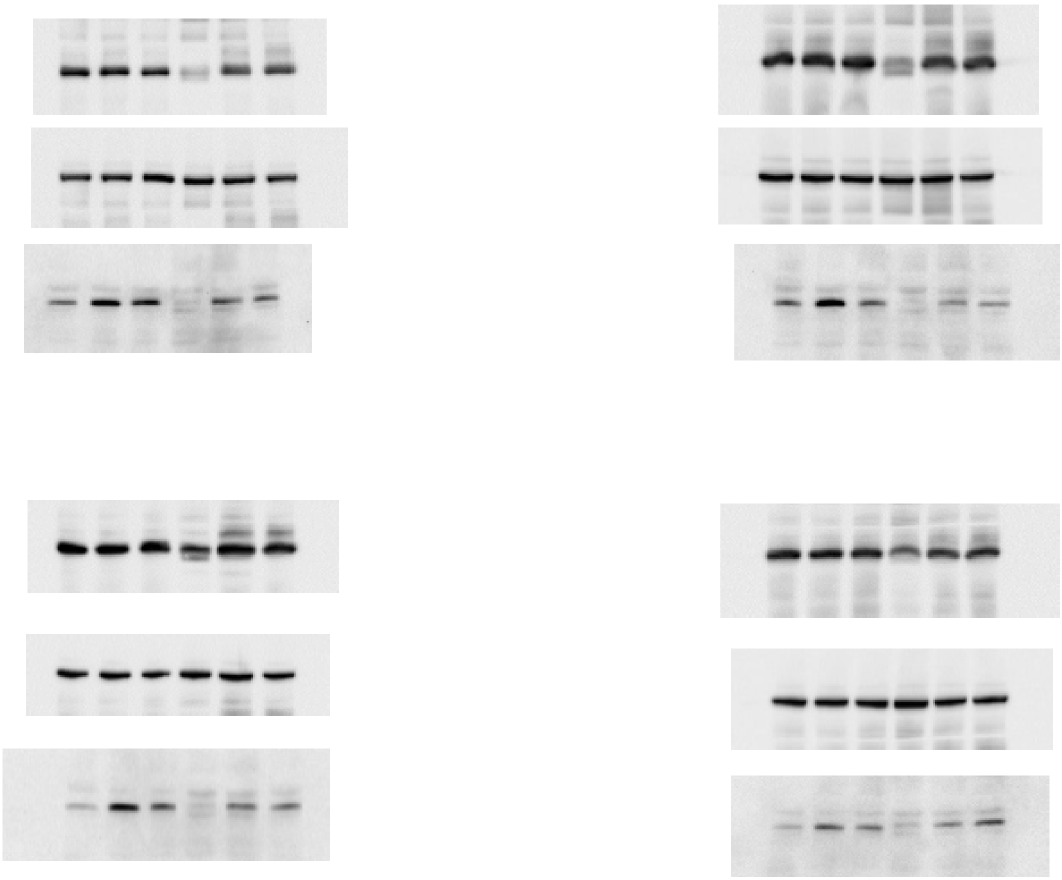

5C

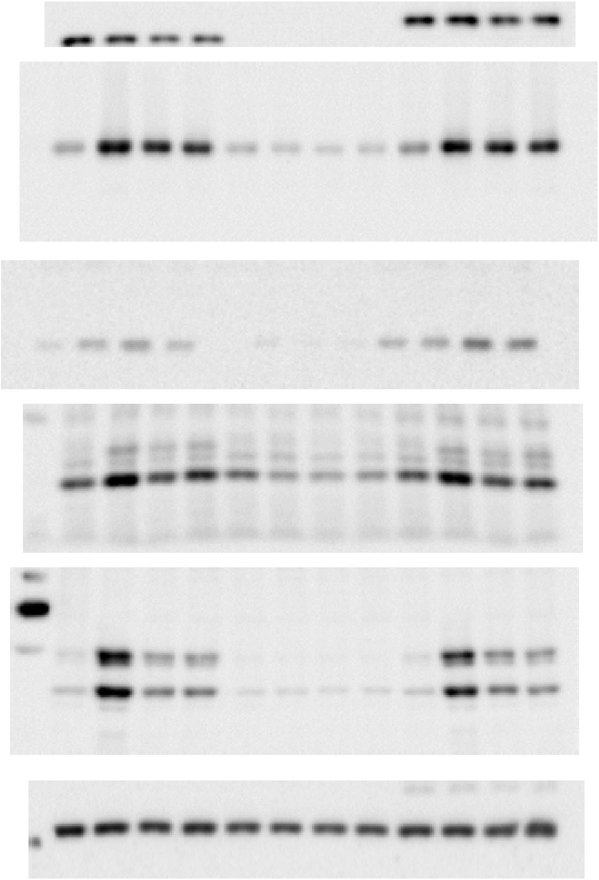

6A

Figure S8. Whole Western Blots

Table S1. Antibodies and plasmids.

**Primary antibody (clone)**	**Species**	**Supplier**
anti-TRAF6 (EP591Y)	rabbit mAb	Abcam
anti-IKKε (72B587)	mouse mAb	Abcam
anti-Vinculin (V9131)	mouse mAb	Sigma
anti-HA (3F10)	rat mAb	Sigma
anti-Flag (M2)	mouse mAb	Sigma
anti-Myc (9E10)	mouse mAb	Sigma
anti-p62 (D3)	mouse mAb	Santa Cruz
anti-phospho p65 (Ser536) (93H1)	rabbit mAb	Cell signaling
anti-phospho I◎B◎ (14D4)	rabbit mAb	Cell signaling
anti-phospho p38 (#9211)	rabbit pAb	Cell signaling
anti-phospho JNK (81E11)	rabbit mAb	Cell signaling
anti-Myc (9E10)	mouse mAb	Sigma
anti-β-Tubulin (Tub2.1)	mouse mAb	Sigma

anti-β-Actin	rabbit pAb	Abcam
IgG control	mouse pAb	Santa Cruz
IgG control	rabbit pAb	Santa Cruz

**Secondary antibody (clone)**	**Conjugated to**	**Supplier**
goat-anti-mouse IgG	HRP	Dianova
goat-anti-rabbit IgG	HRP	Dianova
goat-anti-rat IgG	HRP	Dianova

**Plasmid**	**Origin**	**Reference**
Myc-IKKε	Dr. Chariot, Liege	PMID: 12133833 [1]
Flag-IKKε WT	Schmitz lab	PMID: 20507904 [2]
Flag-IKKε KD	Schmitz lab	PMID: 20507904 [2]
Flag-TRAF6 and mutants	Dr. Darnay, Houston	PMID: 17135271 [3]
His_6-HA-ubiquitin	R. Baer, New York	PMID: 15166217 [4]
$(HA)_2$-TRAF6 WT	this study	
$(HA)_2$-TRAF6 P-def	this study	
$(HA)_2$-TRAF6 P-mim	this study	
$(HA)_2$-TRAF6 C70A	this study	
px459	Dr. Feng Zhang lab	Addgene
px459-TRAF6	this study	

**Oligo name**	**Sequence (5´ to 3´)**
px459-TRAF6-f	caccgtgggtggaactgccagcacg
px459-TRAF6-r	aaaccgtgctggcagttccacccac

References

1. Chariot A, Leonardi A, Muller J, Bonif M, Brown K, Siebenlist U. Association of the adaptor TANK with the I kappa B kinase (IKK) regulator NEMO connects IKK complexes with IKK epsilon and TBK1 kinases. *J. Biol. Chem*. **2002**, 277, 37029-37036, doi:10.1074/jbc.M205069200.
2. Moreno R, Sobotzik JM, Schultz C, Schmitz ML. Specification of the NF-kappaB transcriptional response by p65 phosphorylation and TNF-induced nuclear translocation of IKK epsilon. *Nucleic Acids Res*. **2010**, *38*, 6029-6044, doi:10.1093/nar/gkq439.
3. Lamothe B, Besse A, Campos AD, Webster WK, Wu H, Darnay BG. Site-specific Lys-63-linked tumor necrosis factor receptor-associated factor 6 auto-ubiquitination is a critical determinant of I kappa B kinase activation. *J. Biol. Chem*. **2007**, *282*, 4102-4112, doi:10.1074/jbc.M609503200.
4. Choudhury AD, Xu H, Baer R. Ubiquitination and proteasomal degradation of the BRCA1 tumor suppressor is regulated during cell cycle progression. *J. Biol. Chem*. **2004**; *279*, 33909-33918, doi:10.1074/jbc.M403646200.
